# Conditional expression explains molecular evolution of social genes in a microbe

**DOI:** 10.1038/s41467-019-11237-2

**Published:** 2019-07-23

**Authors:** Janaina Lima de Oliveira, Atahualpa Castillo Morales, Balint Stewart, Nicole Gruenheit, Jennifer Engelmoer, Suzanne Battom Brown, Reinaldo A. de Brito, Laurence D. Hurst, Araxi O. Urrutia, Christopher R. L. Thompson, Jason B. Wolf

**Affiliations:** 1 Milner Centre for Evolution and Department of Biology and Biochemistry, University of Bath, Claverton Down, Bath, BA2 7AY UK; 2 Centre for Life’s Origins and Evolution, Department of Genetics, Evolution and Environment, University College London, Darwin Building, Gower Street, London, WC1E 6BT UK; 3 Faculty of Life Sciences, Michael Smith Building, University of Manchester, Oxford Rd, Manchester, M13 9PT UK; 4 Departamento de Genética e Evolução, Universidade Federal de São Carlos, São Carlos, 13565-905 Brazil; 5 Institute of Ecology, UNAM, Mexico City, 04510 Mexico

**Keywords:** Molecular evolution, Social evolution, Evolutionary genetics

## Abstract

Conflict is thought to play a critical role in the evolution of social interactions by promoting diversity or driving accelerated evolution. However, despite our sophisticated understanding of how conflict shapes social traits, we have limited knowledge of how it impacts molecular evolution across the underlying social genes. Here we address this problem by analyzing the genome-wide impact of social interactions using genome sequences from 67 *Dictyostelium discoideum* strains. We find that social genes tend to exhibit enhanced polymorphism and accelerated evolution. However, these patterns are not consistent with conflict driven processes, but instead reflect relaxed purifying selection. This pattern is most likely explained by the conditional nature of social interactions, whereby selection on genes expressed only in social interactions is diluted by generations of inactivity. This dilution of selection by inactivity enhances the role of drift, leading to increased polymorphism and accelerated evolution, which we call the Red King process.

## Abstract (web summary)

Genetic diversity in social genes is expected to be shaped by conflict. Here, the authors show that in *Dictyostelium discoideum*, social genes in fact exhibit diversification patterns consistent with relaxed purifying selection, likely due to their expression only in intermittent social generations.

## Introduction

The social environment can have profound effects on fitness and, consequently, constitutes an important source of selection^1^. Social environments likely provide their most significant contribution to selection when interactions are characterized by conflict and competition. This is because antagonism can generate a persistent, constantly changing source of selection, where social traits evolve in response to selection, and in turn change the nature of selection itself. To date, research has largely focused on understanding how such conflict-driven selection affects the evolution of social traits, with the implied assumption that the genes underlying social traits (i.e., social genes)^2^ would show similar patterns and processes. Consequently, despite our sophisticated understanding of social trait evolution, we still have a limited understanding of how conflict impacts the social genes themselves^3–7^. This is perhaps surprising given that the patterns of molecular evolution at social genes could help us better understand the key genes behind social traits, the nature of selection arising from social interactions, and the relative importance of different conflict-driven processes in shaping social evolution.

The relentless selection resulting from social conflict is analogous to the Red Queen process, where competition in the ecological environment generates persistent counter-evolutionary change in interacting parties^2,8^. The role of the Red Queen process in social evolution depends on how the traits expressed by interactants determine their fitness through the interaction^1^. Likewise, the consequences of these interactions on molecular evolution will depend on the relationship between sequence variation at social genes and the properties of the social traits. One possibility is that selection drives constant evolutionary change in social traits because of reciprocal counter-evolution of competitive strategies akin to the Escalatory Red Queen process^8^. This process would presumably proceed as a series of selective sweeps of advantageous mutations at the associated social genes, reducing levels of polymorphism and increasing the rate of evolutionary divergence. Alternatively, success in social interactions may depend on the specific properties of the opponent or context in which competition occurs, which could result in a scenario where different social traits, and hence genetic variants at associated social genes, are favored in different social contexts. Such non-transitivity is akin to the Fluctuating Red Queen process^8^, where frequency dependent selection maintains genetic variation that underlies alternative strategies, which would be manifested as a signature of balancing selection^9^.

Although the Red Queen processes can have marked consequences for patterns of trait and molecular evolution, conflict can also potentially result in the opposite outcome, where evolutionary change is halted by the emergence of an evolutionarily stable strategy (ESS)^10^. Populations at the ESS would experience optimizing selection to remain at the ESS, resulting in evolutionary stasis with purifying selection on associated social genes. This purifying selection is expected to lead to low levels of polymorphism and divergence, which are at direct odds with the predictions of the Fluctuating and Escalatory Red Queen processes^8^. It is therefore possible to differentiate between contradictory predictions of conflict driven selection by evaluating signatures of selection on social genes, thus providing important insights into the nature and consequences of selection arising from social interactions.

Investigations into the form and consequences of selection generated by conflict must necessarily also consider the potentially confounding role of the random processes of drift and mutation^11–13^. Although this is true for all types of genes, it is particularly critical for social genes in organisms that are facultatively social or those that otherwise only rarely encounter conflict. Such conditionality could diminish the impact of selection and enhance the role of drift^12^. We refer to this scenario as the Red King process with reference to the character from Lewis Carroll’s “*Through the Looking Glass*”. Unlike the Red Queen, who is known for her active role throughout Carroll’s story, the Red King is asleep through most of the book. Hence, we invoke the Red King to refer to his defining characteristic of inactivity, with the Red King process capturing the consequences of periodic inactivity of a gene (or more generally, a lack of selection) that dilutes the relative impact of selection (which differs from the use of the term to describe mutualistic coevolution^14^). For example, the Red King process can be important under a variety of scenarios when gene expression is limited to a fraction of generations or a subset of all individuals^12^.

Although both the Red King process and the Fluctuating Red Queen process can potentially result in elevated polymorphism, they typically differ in the specific signatures they predict. For example, the Red King process predicts an overall shift toward neutrality^12^, whereas the Fluctuating Red Queen process is predicted to result in elevated functional variation underlying adaptive alternatives^8^. Likewise, the Red King process can result in an elevated rate of fixation of slightly deleterious mutations by drift. Because most slightly deleterious mutations are nonsynonymous, greater fixation of nonsynonymous variation could resemble the pattern of positive selection favouring new strategies predicted for the Escalatory Red Queen process. However, diluted selection under the Red King process should allow for elevated levels of segregating deleterious polymorphism^12^, whereas the selective sweeps of the Escalatory Red Queen process would lead to lower levels of polymorphism^8^. Thus, in order to differentiate the impacts of different processes on molecular evolution we need to consider the joint impacts of selection and drift on patterns of divergence and polymorphism at social genes. Here we address this problem by examining evolutionary signatures at genes potentially shaped by conflict in the social microbe *Dictyostelium discoideum*.

*D. discoideum* provides a powerful model system for evaluating the joint consequences of social conflict^15^ and the Red King process on molecular evolution at social genes. *D. discoideum* live as single celled individuals in the soil, but aggregate together in response to starvation to form a multicellular slug that eventually forms a fruiting body that aids spore dispersal^16^ (Supplementary Figure 1A). Construction of a functioning fruiting body requires cooperation among cells, with some cells killed to form the stalk, whereas others form viable spores. When multiple genotypes co-aggregate, this differentiation into stalk and spores is expected to generate conflict over representation in spores^15^. Because amoebae will live and reproduce continuously in the vegetative stage as long as there is adequate food, the social (multicellular) stage represents a conditional strategy, with each generation of social development being preceded by many generations of vegetative growth (Supplementary Figure 1A). As a result, genes associated with the social stage are expected to experience conditional selection, providing an opportunity for the Red King process to shape patterns of evolution at genes expressed during that stage.

Previous analyses of social traits in this system have demonstrated enormous phenotypic diversity in traits associated with the social stage^17–19^. This degree of phenotypic diversity suggests that evolutionary processes promote variation at social genes. However, a detailed analysis of social strategies suggests that there is potentially a single ESS, with facultative cooperation and cheating based on relatedness^20^, and therefore the presence of diversity is also potentially consistent with the expectations of relaxed purifying selection (which favors a single optimal strategy) under the Red King process. Previous attempts to characterize patterns of molecular evolution at social genes in *D. discoideum* have led to seemingly contradictory reports of signatures of balancing selection^6^, accelerated evolution, and purifying selection^7^ depending on the set of genes being considered. Therefore, to understand how social interactions have shaped gene sequence evolution, we implement an integrative approach using large-scale gene expression, functional genomics, and genome sequence data from 67 natural strains. Our analyses provide strong support for a unified perspective, with all evidence consistent with the conclusion that social genes experience a similar overall pattern of selection as other classes of genes. However, because the expression of some social genes is restricted to the social stage, the patterns of molecular evolution manifest a signature of diluted selection owing to the Red King process.

## Results

### Identification of social genes

To understand broad-scale processes shaping molecular evolution at social genes we have used four different, but complementary, approaches to identify sets of social genes. For ease, we have named these sets sociality, chimerism, antagonism, and cheater genes. For comparison, we have also identified appropriate sets of control genes.

Sociality genes are defined as those with expression restricted to the social stage (which corresponds to the period of aggregation and multicellular development) (Supplementary Figure 1A). Because sociality genes are only expressed in social stages, their evolutionary signatures should reflect the overall selective impact of social interactions. To identify sociality genes, we used large-scale transcriptome data from vegetative growth on bacteria or in liquid culture^21–23^ and high-resolution transcriptome data from multiple stages of the social cycle, from starvation to the formation of mature fruiting bodies^21^. We calculated the Index of Social Expression (ISE)^24^ for each gene by comparing the expression at social stages (hour 1–24) to the expression at both social and single celled vegetative stages (hour 0). As expected, we find a clear discontinuity in the distribution of ISE values (Supplementary Figure 2A). 1650 genes exhibited a high bias in expression to social stages (ISE > 0.9; i.e., >90% of its expression concentrated in social stages), which we consider to be the set of sociality genes. Signatures of selection in sociality genes were compared against all genes with some level of expression in the full transcriptome data set (i.e., all genes with some measured level of expression at any timepoint in development or in the vegetative stages).

Sociality genes were found to be expressed at remarkably low levels at the vegetative stage (median = − 0.64 log_10_TPM, Supplementary Figure 2B), demonstrating that they are conditional to social development and not simply upregulated at this stage. Sociality genes show relatively high expression levels when expressed in the social cycle (Supplementary Figure 2C). As expected, they are overrepresented for GO categories related to development (Supplementary Table 1). They are also enriched for genes without biological process annotation (permutation test *FDR*-corrected *p* < 10^−4^, Supplementary Table 1), which may reflect lack of conservation and orthology with characterized genes, potentially reflecting rapid evolution.

Chimerism genes are defined as those significantly upregulated in chimeric aggregations in comparison to clonal aggregations. Chimeric development is potentially characterized by conflict, and hence these genes might show signatures of conflict driven evolution^7^. Differential expression analysis revealed 190 genes, which are enriched in GO categories mostly related to functions associated with vegetative growth (Supplementary Table 2). Evolutionary patterns at these chimerism genes were compared against all genes expressed in the same contexts (i.e., all genes showing some level of expression under either condition).

Antagonism genes are defined as those genes that are preferentially expressed in cells destined to become the stalk or spores. They are potentially shaped by antagonism because cell fate choice in *D. discoideum* determines, which cells end up having zero direct fitness by providing the dead stalk and which get the direct benefit by producing spores^25–27^. Antagonism genes were identified as those that show significant differential expression in the cell populations in slugs that lead to the formation of the stalk (prestalk genes) and the spores (prespore genes)^7,23^ (Supplementary Figure 1C). A total of 1901 genes show significant differential expression in either of these regions (prespore = 903 and prestalk = 998)^7^. Antagonism genes are enriched in GO categories related to cell membrane, extracellular region and cytoskeleton (Supplementary Table 3). Signatures of selection in these genes were compared with the background of all genes expressed in prespore and prestalk cells^23^.

A set of 99 cheater genes have previously been identified experimentally. When these genes are mutated, strains become overrepresented among the spores when mixed with wild type cells^28^ (Supplementary Figure 1D). Evolutionary signatures at these genes were compared with the rest of the protein-coding genes in the genome. GO term analysis revealed that cheater genes are overrepresented in only one category of biological process: social behavior (Supplementary Table 4), which may be tautological because it is based on the mutagenesis screen used to identify these genes.

There is little overlap between the sociality, cheater and chimerism sets of social genes (Table 1). Chimerism genes are not a subset of the sociality genes (Table 1), and their mean ISE value is not significantly different from the rest of the genome (ISE_Chimerism_ = 0.51, ISE_Background_ = 0.54; *t* test: *FDR*-corrected *p* = 0.084). Chimerism genes are actually significantly enriched for genes with the peak of maximum expression during vegetative growth (expected: 79; observed: 104; Chi square test: *p* < 0.0003) and are enriched in GO categories mostly related to functions that are associated with vegetative growth (Supplementary Table 2). Moreover, there is no significant overlap between cheater and sociality genes (Table 1). Although eight of the 99 cheater genes are expressed at such low levels during vegetative and developmental stages (across all sequenced RNA pools) that we cannot characterize their expression profile, the mean ISE for the remaining 91 genes is 0.53, which is not significantly different from all other genes (ISE_Cheater_ = 0.54, ISE_Background_ = 0.53; *t* test: *FDR*-corrected *p* = 0.740). We also do not find an overlap between cheater and chimerism genes (Table 1). In contrast, there is a significant enrichment in the overlap between antagonism genes and both sociality and chimerism genes (Table 1). This enrichment presumably reflects the fact that the antagonism genes were identified based on differential expression in slugs, and hence temporally overlap with the sociality and chimerism genes (both identified from expression in social stages). This idea is supported by the fact that the antagonism genes show a significantly higher ISE than their respective background genes (ISE_Antagonism_ = 0.63, ISE_Background_ = 0.53; *t* test: *FDR*-corrected *p* < 10^−48^). This difference appears in both the prespore (ISE_Prespore_ = 0.66, ISE_Background_ = 0.53; *t* test: *FDR*-corrected *p* < 10^−44^) and prestalk (ISE_Prestalk_ = 0.59, ISE_Background_ = 0.54; *t* test: *FDR*-corrected *p* < 10^−8^) subsets, with a significantly higher index in the prespore set compared with prestalk (*t* test: *FDR*-corrected *p* < 10^−7^).

**Table 1:** Social genes in the amoeba *D. discoideum*

	Sociality	Chimerism	Antagonism	Cheater
Sociality	─	22	507**	9
Chimerism	26	─	44*	2
Antagonism	261	30	─	15
Cheater	13	1	14	─

Sociality genes are effectively expressed in the facultatively expressed social cycle, as measured by an index of social expression (see Methods). Chimerism genes are those differentially expressed in chimeras compared with clonal development, specifically at the slug stage. Antagonism is a group formed by previously identified genes differentially expressed in prespore and prestalk cells^7,23^. Cheater genes were previously characterized from mutagenesis screenings to identify mutants with defective behavior^28^. Significance for the overlaps between each pair of gene categories was obtained by chi-square tests. Cell entries give the observed (upper diagonal) and expected (lower diagonal) values, with significant deviations from expected (*FDR-*corrected *p* < 0.05) being indicated by bold observed values. * indicates *FDR-*corrected *p* < 0.05, whereas ** indicates *p* *<* 10^−70^

### Sociality and antagonism genes harbor increased diversity

**Fig. 1:**
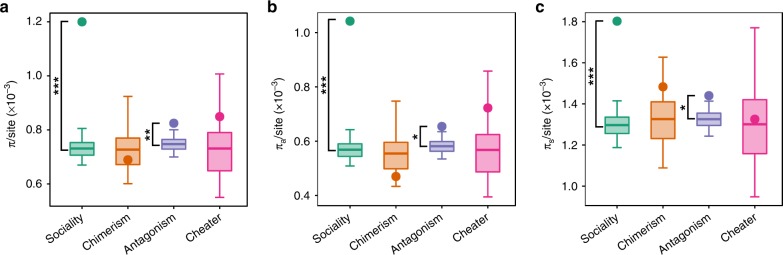
Polymorphism in social genes. Average estimates of nucleotide diversity per site (*π*/site) for CDS **a**, nonsynonymous **b**, and synonymous **c** sites for each group of genes (points) were compared with randomization distributions (boxplots). The middle line, bottom, and top of the box show the expected mean, 25th and 75th percentiles, respectively; whiskers present the 95% confidence interval of the distributions. Randomization distributions were generated for each group of social genes by generating a set of 10,000 random groups of genes of size *N* (where *N* corresponds to the number of genes in the particular group of social genes being tested). Randomization was done separately for each group of social genes by sampling from a set that contains that group of social genes and its corresponding background set of genes. Two-tailed *p* values are defined as the probability of obtaining a mean as extreme as the observed only owing to chance. Significance after *FDR* correction: *p* < 0.05 *; *p* < 0.01 **; *p* < 0.001 ***. Source data are provided as a Source Data file

To compare patterns of polymorphism in the four sets of social genes to their respective background gene pools, we generated genome sequence data from a set of 47 wild strains and combined these with sequence data from 20 published wild-strain genomes^29^. Sequence information was obtained for 12,809 protein-coding sequences. Both sociality and antagonism genes harbour significantly more variation compared with their background expectation, whether estimated by average nucleotide diversity per site (*π*/site; Fig. 1) or the number of SNPs per site (SNP/site; Supplementary Table 5). Variation at sociality and antagonism genes is greater across the entire CDS, including both nonsynonymous and synonymous sites. The pattern of overall elevated polymorphism is consistent with the Red King process, where signatures of relaxed selection are manifested at both synonymous and nonsynonymous sites. It is inconsistent with the Fluctuating Red Queen process, where we expect signatures of balancing selection at nonsynonymous sites but not synonymous sites. The fact that both antagonism genes and sociality genes show elevated polymorphism raises the possibility that the shared patterns are caused by overlapping set of genes. It is, therefore, possible that either the elevated polymorphism at sociality genes is, at least in part, caused by the sharing of antagonism genes, or, likewise, that the patterns at antagonism genes are explained by their content of sociality genes. Indeed, we find that a permutation test where we randomly resample genes containing the same proportion of sociality genes as we see in the set of antagonism genes produces an almost identical pattern of polymorphism as we observe in the data. In contrast, randomly resampling sets of genes with a similar proportion of antagonism genes as we observe in the sociality genes fails to account for their levels of polymorphism, giving much lower predicted values for all polymorphism parameters compared with what we observe (Supplementary Table 6). This strongly supports the conclusion that the content of sociality genes explains the elevated polymorphism at antagonism genes (and not vice versa). This conclusion is supported by the fact that, if we remove the subset of sociality genes from within the set of antagonism genes, the remaining set of antagonism genes no longer shows a difference in polymorphism compared with their background (Supplementary Table 7). In contrast, removing the subset of antagonism genes from the sociality genes has no impact on the patterns of polymorphism at the sociality genes (Supplementary Table 8). Therefore, we focus on investigating the evolutionary patterns at sociality genes to understand the patterns we observe since the process causing elevated polymorphism at sociality genes explains the appearance of elevated polymorphism at antagonism genes.

The pattern of polymorphism observed at sociality genes could reflect infrequent selection owing to their conditional expression. Alternatively, these genes could experience relaxed selection if they are dispensable for social development. To distinguish between these possibilities, we analyzed the signatures of selection on subsets of sociality genes for which there is additional evidence to support their importance during the developmental cycle. First, we restricted our analysis to sociality genes previously identified as exhibiting evolutionarily conserved sequences and expression profiles^30^, which are likely to be functionally important for normal development. Second, we restricted our analyses to sociality genes with the highest levels of expression, and thus should not simply reflect relaxation of transcriptional control during development. When we analyze genes with conserved expression^30^ (Supplementary Table 9), or when we have removed the genes with the lowest expression (Supplementary Table 10), we still find that the same pattern of elevated nonsynonymous and synonymous polymorphism as observed for the complete set of sociality genes. This provides strong support that it is associated with expression being limited to the social phase.

Finally, we tested whether the elevated polymorphism at sociality genes is a result of them having a higher ISE rather than the fact that they are conditionally expressed. We found no correlation between the ISE values and levels of polymorphism (Supplementary Table 11). To further test this idea and better understand the broader impacts of conditional expression and the Red King process, we also identified a set of conditionally expressed non-social protein-coding genes, which have zero expression at all time points (but are presumably expressed under some other conditions). We expect these genes to experience diluted selection relative to non-conditional genes (which are represented by the non-sociality genes that are expressed throughout the life cycle). Again, we see the same pattern of overall elevated polymorphism (both nonsynonymous and synonymous) in these genes (Supplementary Table 12). Together, these data suggest that conditional expression of sociality genes leads to a pattern of elevated polymorphism consistent with the Red King process.

### Higher variation at sociality genes reflects weak selection

To further differentiate between alternative explanations for the pattern of elevated variation at sociality genes, we examined the distribution of variation and the type of variation present. We calculated average Tajima’s *D* values^31^ for each set of social genes, where negative values indicate an excess of low frequency variants (presumably reflecting erosion of variation by purifying selection or selective sweeps), and positive values an excess of intermediate frequency variants (reflecting maintenance of variation by balancing selection). The average *D* for sociality genes is negative for the whole coding sequence, as well as at nonsynonymous and synonymous sites when they are considered separately, but is not significantly different from that expected from the background genes (Table 2). This pattern is inconsistent with that expected under balancing selection and consistent with the expectation under either purifying selection or recent selective sweeps. This finding is supported by results from other neutrality tests, either using information from the site frequency spectrum (Fu & Li’s F* and D*) or linkage disequilibrium statistics (Wall’s *Q* and *B*; Supplementary Table 13). To address the possibility that a subset of sociality genes experiences balancing selection, inflating the average polymorphism level for the group, we used two approaches. First, we tested whether sociality genes are enriched for genes evolving under balancing selection, using three different thresholds of *D* to define a signature of balancing selection (*D* = 2, *D* = 1.5, and *D* = 1). We find no evidence of such overrepresentation (Supplementary Table 14). We also tested whether hyper-variable outliers among the sociality genes could be inflating the overall pattern of elevated nucleotide diversity within this group. For this we removed the 13 sociality genes evolving under balancing selection (*D* > 2), and still find that sociality genes exhibit significant higher levels of polymorphism (Supplementary Table 15).

**Table 2:** Tajima’s *D* for social genes. Expected values and the respective two-tailed *p* values were obtained by a randomization process

Sites	Group	Expected	Observed	*p* (*FDR*)
CDS	Sociality	−0.651	−0.709	0.085
	Chimerism	−0.650	−0.660	0.938
	Antagonism	−0.651	−0.663	0.739
	Cheater	−0.653	−0.724	0.846
Nonsynonymous	Sociality	−0.634	−0.694	0.085
	Chimerism	−0.634	−0.635	0.993
	Antagonism	−0.634	−0.674	0.232
	Cheater	−0.635	−0.715	0.739
Synonymous	Sociality	−0.467	−0.484	0.739
	Chimerism	−0.466	−0.502	0.739
	Antagonism	−0.466	−0.444	0.739
	Cheater	−0.468	−0.523	0.739

For each group of social genes, we generated a set of 10,000 random groups of size *N* (where *N* is the number of genes in that particular group) sampled from a set that contains that group of social genes and its corresponding background set of genes. Two-tailed *p* values are defined as the probability of obtaining a mean as extreme as the observed only owing to chance after *FDR* correction for multiple tests

To further differentiate between explanations for the elevated polymorphism at sociality genes, we focused on classes of segregating variation that are presumably deleterious. For this, we examined the presence of two special types of mutations: SNPs that introduce a stop codon, and mutations that correspond to complete or partial gene deletion (which is characterized by presence-absence variation; PAV). We find that sociality genes are enriched for genes with both types of deleterious mutations (Table 3), which is consistent with relaxed purifying selection, thus providing further support for the Red King process. Interestingly, we also find that antagonism genes have a significant dearth of presence/absence variation, suggesting that they may be enriched for essential genes.

**Table 3:** Enrichment analysis of the number of social genes carrying at least one mutation that introduces a stop codon or results in a partial deletion (presence/absence variation)

Class of mutations	Group	Observed	CI		*p* (*FDR*)
Stop codon gain	Sociality	79	46	72	**0.022**
	Chimerism	5	2	11	> 0.05
	Antagonism	11	4	15	> 0.05
	Cheater	9	1	8	> 0.05
Presence/Absence	Sociality	12	2	10	**0.042**
	Chimerism	0	0	2	> 0.05
	Antagonism	0	9	24	**0.002**
	Cheater	1	0	3	> 0.05

We used a randomization procedure to test whether each of the five groups of genes contained an excess of genes carrying these types of deleterious mutations. For each group of genes, we generated a set of 10,000 random groups of size *N* (where *N* is the number of genes in that particular group) sampled from a set that contains that group of social genes and its corresponding background set of genes. In each randomization we counted the number of genes that contained each type of deleterious mutation and used the distribution of the counts across randomizations to calculate the confidence intervals (2.5th to 97.5th percentiles) and *p* values. Significant *p* values after *FDR* correction for multiple tests are highlighted in bold (*FDR* *<* 0.05)

### All classes of social genes reflect purifying selection

Analyses of the patterns of polymorphism provide only a partial picture of the nature of selection because different evolutionary processes can potentially result in similar levels of standing variation. Therefore, we complemented our analyses of segregating polymorphism with two analyses that draw on patterns of evolutionary substitution to capture patterns of selection in deeper evolutionary time. First, we compared levels of polymorphism to fixed differences in a highly divergent *D. discoideum* strain from Mexico (OT3A). Second, we characterized the rate of protein sequence evolution by comparing the reference genome^32^ to this divergent strain.

Using polymorphism data from the 67 natural *D. discoideum* strains and the divergent OT3A, we compared the number of segregating and fixed differences at each gene using the McDonald–Kreitman test (MKT)^33^. We found 47 genes that harbour a significant excess of nonsynonymous substitutions (*D*_*n*_) and 94 showing an excess of nonsynonymous polymorphism (*P*_*n*_). We next tested whether either of these classes of genes is enriched in any of the four groups of social genes in comparison to that expected for their comparable set of background genes. In sociality genes, we observe an underrepresentation of genes evolving under positive selection, suggesting a restricted role of adaptive evolution in this group (Supplementary Table 16). For all other classes of genes, we find no significant overrepresentation of genes evolving under positive or balancing/purifying selection.

**Fig. 2:**
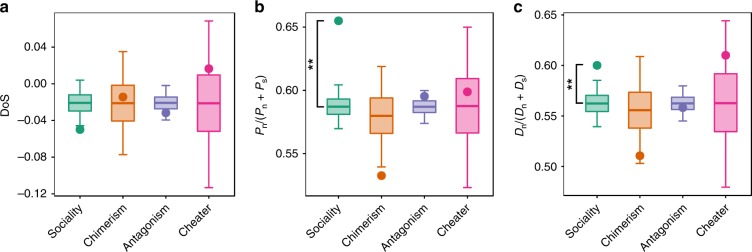
The Direction of Selection (*DoS*) statistics for social genes. Given is the *DoS* value **a**, where *DoS* = (*D*_*n*_/(*D*_*n*_ + *D*_*s*_))-(*P*_*n*_/(*P*_*n*_ + *P*_*s*_)), and the value of each of its component parts: the proportion of polymorphisms *(P*_*n*_/(*P*_*n*_ + *P*_*s*_)) **b** and substitutions (*D*_*n*_/(*D*_*n*_ + *D*_*s*_)) **c** that are nonsynonymous. Average estimates for each group of genes (points) were compared with randomization distributions (boxplots). The middle line, bottom, and top of the box show the expected mean, 25th and 75th percentiles respectively; whiskers present the 95% confidence interval of the distributions. Randomization distributions were generated for each group of social genes by generating a set of 10,000 random groups of genes of size *N* (where *N* corresponds to the number of genes in the particular group of social genes being tested). Randomization was done separately for each group of social genes by sampling from a set that contains that group of social genes and its corresponding background set of genes. Two-tailed *p* values are defined as the probability of obtaining a mean as extreme as the observed only owing to chance. Significance after *FDR* correction: *p* < 0.05 *; *p* < 0.01 **; *p* < 0.001 ***. Source data are provided as a Source Data file

The MKT, which is based on a significant excess of either *P*_*n*_ or *D*_*n*_, provides a conservative analysis and may not reveal subtle quantitative differences in evolutionary signatures. Therefore, we complemented the MKT with the Direction of Selection statistic: *DoS* = *D*_*n*_/(*D*_*n*_ + *D*_*s*_) − *P*_*n*_/(*P*_*n*_ + *P*_*s*_)^34^. This approach provides a quantitative measure of the pattern of substitution relative to polymorphism, with zero indicating neutrality, positive values indicating adaptive evolution, and negative values indicating balancing selection or segregation of slightly deleterious variation. The expected *DoS* values for all classes of social genes are negative, and none of the classes are significantly different than expected compared with their background (Fig. 2). This finding is consistent with the hypothesis that all classes of genes experience a similar form of selection as the background (which is captured by their similar *DoS* values). Although the average *DoS* values for the classes of social genes do not differ from their respective backgrounds, we find that the individual components of the *DoS*—the proportion of substitutions (*D*_*n*_/(*D*_*n*_ + *D*_*s*_)) and polymorphisms (*P*_*n*_/(*P*_*n*_ + *P*_*s*_)) that are nonsynonymous—differ from the background for the sociality genes. This variation in the components of the *DoS* reflects variation in the strength (not form) of selection (since the form of selection is captured in the overall *DoS* value). For the sociality genes, the averages for both components of *DoS* are higher, indicating both elevated variation and divergence. This pattern is inconsistent with the hypothesis that balancing selection maintains polymorphism. Instead, the overall pattern suggests weaker purifying selection on these genes, which leaves more deleterious mutational variation segregating in the population and results in an increased probability of mutations eventually reaching fixation. These same patterns are seen for sociality genes when analyzed in the subset where we remove those with the lowest expression (Supplementary Table 10). We also see these same patterns at the conditional genes (Supplementary Table 12), suggesting they share a similar profile of selection as the sociality genes. For both the cheater and antagonism genes, neither of the *DoS* components differ from the background values. Taken together, the quantitative *DoS* values suggest that all classes of social genes predominantly show patterns consistent with purifying selection (which is captured by the negative *DoS* values), but vary in the relative intensity of selection (which is captured in the variation we see in the components of the *DoS*, which vary across gene classes, but always deviate from the expected value in the same direction within a gene class).

**Fig. 3:**
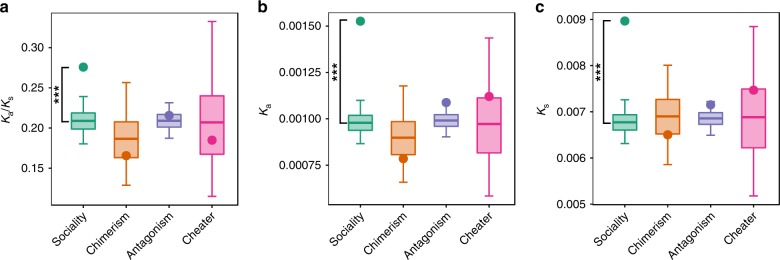
Evolutionary rates at social genes. Given is the evolutionary rates **a**, and rates of substitution at nonsynonymous (*K*_*a*_) **b** and synonymous (*K*_*s*_) sites **c**. Substitutions represent changes compared with the sequence of a divergent strain, OT3A, from Mexico. Average estimates for each group of genes (points) were compared with randomization distributions (boxplots). The middle line, bottom, and top of the box show the expected mean, 25th and 75th percentiles, respectively; whiskers present the 95% confidence interval of the distributions. Randomization distributions were generated for each group of social genes by generating a set of 10,000 random groups of genes of size *N* (where *N* corresponds to the number of genes in the particular group of social genes being tested). Randomization was done separately for each group of social genes by sampling from a set that contains that group of social genes and its corresponding background set of genes. Two-tailed *p* values are defined as the probability of obtaining a mean as extreme as the observed only owing to chance. Significance after *FDR* correction: *p* < 0.05 *; *p* < 0.01 **; *p* < 0.001 ***. Source data are provided as a Source Data file

Rates of protein evolution were calculated from pairwise gene alignments using the reference genome^32^ and the strain OT3A. The number of nonsynonymous substitutions per nonsynonymous site (*K*_*a*_) was compared with the number of synonymous substitutions per synonymous site (*K*_*s*_) to identify signatures of selection. The ratio *K*_a_/*K*_s_ is expected to be ~1 if nonsynonymous sites are nearly neutral, >1 if they are under positive selection, and <1 if they are under purifying selection^35^. We identified 5509 protein-coding orthologues between this pair of lineages, and estimated *K*_*a*_, *K*_*s*_, and their ratio (which does not include those for which the ratio could not be calculated, such as when synonymous sites are saturated). We then tested whether the four classes of social genes differed from their respective backgrounds. For all sets of social genes, the average *K*_*a*_/*K*_*s*_ is < 1, but patterns varied across the classes (Fig. 3). Cheater, chimerism, and antagonism genes do not differ from their respective backgrounds for any parameter (*K*_*a*_, *K*_*s*_, or *K*_*a*_/*K*_*s*_). In contrast, sociality genes show increased substitution rates at both nonsynonymous and synonymous sites. Because nonsynonymous divergence shows a larger difference than synonymous divergence, the overall rate of evolution (*K*_*a*_/*K*_*s*_) is also elevated compared with the background rate. These same patterns are seen for sociality genes when we remove those with the lowest expression (Supplementary Table 10). Similarly, when the subset with conserved expression profiles is analyzed, both *K*_*a*_ and *K*_*s*_ are significantly different from the background, although the *K*_*a*_/*K*_*s*_ ratio is not, which may reflect more evolutionary constraint on this set of genes; Supplementary Table 9). Although we would expect the Escalatory Red Queen process to lead to accelerated rates of evolution at protein-coding genes, these results are not consistent with the Escalatory Red Queen process expectations because we would not expect the observed elevated rates of evolution at both synonymous and nonsynonymous sites. Hence, these findings support the initial hypothesis that both synonymous and nonsynonymous sites in social genes evolve under purifying selection, but with the Red King process reducing the strength of selection that results in an increased rate of sequence evolution. This conclusion is supported by the analysis of conditional genes (Supplementary Table 12), where we also see elevated rates of both synonymous and nonsynonymous substitutions and an overall rate of evolution (*K*_*a*_/*K*_*s*_) consistent with purifying selection (i.e., *K*_*a*_/*K*_*s*_ < 1).

### The Red King process explains molecular evolution

The patterns of polymorphism and divergence are all consistent with the hypothesis that each class of social genes evolves primarily under purifying selection, with differences being explained by the degree of conditionality of expression, which leads to the Red King process. To test whether the Red King process provides an overall explanation for patterns of molecular evolution, we examined the relationship between the overall degree of conditionality for any gene class (i.e., the proportion of sociality genes in the class) and either the levels of polymorphism or divergence. In this analysis, we use the Red King process as a null hypothesis to predict the properties of the other classes of genes. If any class of genes is simply a random subsample of all genes (with a given proportion of the conditionally expressed sociality genes and the non-conditionally expressed non-sociality genes), then their evolutionary properties should be predictable based on the proportion of genes they contain from these two classes. However, if a class of genes contains a collection of genes that deviate in the form of selection they experience (i.e., the class of genes contains a non-random subsample of all genes), then the class should deviate from this null hypothesis.

**Fig. 4:**
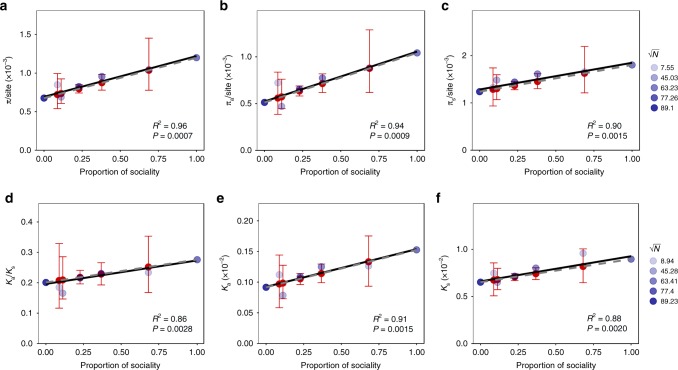
The impact of Red King processes on polymorphism and divergence. The top row of panels shows the relationship between nucleotide diversity in social genes at the full CDS **a**, nonsynonymous **b**, and synonymous sites **c** as a function of the proportion on conditionally expressed (sociality) genes within that group. Similarly, the bottom row of panels shows the relationship between the rates of nonsynonymous **e**, synonymous substitutions **f**, and the rate of protein evolution **d** in groups of genes as a function of the proportion of sociality genes in each group. In each panel the red points indicate the expected value for a group and the red error bars the 95% confidence interval (both calculated from a set of 10,000 permutations). The blue points indicate the observed values for each group of genes, with the darkness of the point being determined by the sample size (based on $\sqrt N$). The dashed line indicates to the expected value of each parameter predicted from the sociality and non-sociality genes. The solid line corresponds to the best fit line from a weighted regression (where weightings are $\sqrt N$) fitted to the observed values. Source data are provided as a Source Data file

To test this idea, we used the sets of genes defined above (chimerism, antagonism, and cheater). In addition, to help illustrate the utility of this perspective, we added three more classes of genes: non-sociality (which show some level of expression in the transcriptome data set used to identify sociality genes, but which are not conditional to the social stage) and two classes of antagonism genes showing differing degrees of expression bias in prestalk versus prespore cells (representing expression biases of 0.8 and 0.9 corresponding to 640 and 109 genes, respectively). These latter two sets of genes contain large percentages of sociality genes, with 38% of the genes in the 0.8 bias class and 69% of the genes in 0.9 bias class being sociality genes. To test whether each class showed patterns consistent with the Red King process, we randomly permuted genes such that the genes assigned to each group contained the same proportion of sociality and non-sociality genes as we observe in the original sets. We then tested whether the observed values of the various evolutionary parameters are significantly different from those expected based on the proportion of sociality genes in that class. We also fitted a weighted regression to characterize the relationship between the proportion of sociality genes in the gene classes and the values for each of the evolutionary statistics (Fig. 4). We find that no class of genes shows a significant deviation from the expected values under the Red King process for any of the evolutionary parameters (Fig. 4). Furthermore, the estimated relationship from the weighted regression model shows that the proportions of sociality genes in each class accounts for the vast majority of the variation in patterns of polymorphism in terms of *π*/site (*R*^2^ = 0.96, 0.94 and 0.90 for the full CDS, nonsynonymous and synonymous sites respectively, with permutation test *FDR*-corrected *p* < 0.002 in all cases; Fig. 4), the rate of synonymous (*K*_*s*_, *R*^2^ = 0.8, permutation test *FDR*-corrected *p* = 0.002) and nonsynonymous (*K*_*a*_, *R*^2^ = 0.91, permutation test *FDR*-corrected *p* < 0.002) divergence, and variation in the rate of nonsynonymous relative to synonymous divergence (*K*_*a*_/*K*_*s*_, *R*^2^ = 0.86, permutation test *FDR*-corrected *p* = 0.003). Taken together, these findings suggest that the variation in evolutionary signatures observed in other studies^6,7^ could reflect artefacts introduced by not accounting for the Red King process as the appropriate null hypothesis. For example, we see significantly elevated values for several of the evolutionary statistics at genes showing biased expression in prestalk and prespore cells (Supplementary Table 17), which could be misinterpreted as evidence that these classes of genes are evolving under some other form of diversifying selection (e.g., balancing selection).

## Discussion

Our analyses provide strong support for a unified evolutionary picture in this system. Social genes primarily experience purifying selection, and where we do find differences in their evolutionary patterns, they are consistent with the Red King process driven by conditional selection. These results emphasize the importance of considering the impact of the Red King process, as well as considering the correct null hypothesis for why genes might show different signatures of selection. Furthermore, they show that the full body of evidence must be evaluated when interpreting patterns of molecular evolution (as incorrect conclusions can be drawn from the results of any single test). These issues may help to explain why patterns of molecular evolution have previously been attributed to social conflict in *D. discoideum*^6,7^, even though we find that they are better explained by the Red King process. For example, elevated rates of adaptive evolution have been reported at a set of genes differentially expressed in chimeric development (compared with clonal development)^7^. The set of genes that this conclusion is based on contains a large percentage of sociality genes (44%, compared with ca. 12% in the relevant background), and hence we would expect elevated polymorphism and divergence. Indeed, the relative counts of synonymous and nonsynonymous substitutions (*D*_*s*_ and *D*_*n*_) and polymorphism (*P*_*s*_ and *P*_*n*_) (scaled in relation to the total sequence length) are consistent with this hypothesis. This is most clearly manifested in the overabundance of synonymous substitutions compared with that expected (which can skew the statistics used to infer the rate of adaptive evolution). Likewise, analyses of genes showing differential expression in cells destined to become stalk (prestalk genes) compared with those destined to become spores (prespore genes), which we have designated as the antagonism genes, have been reported to be more polymorphic than the genomic background^7^. We find that this result matches the expectation for the Red King process, as this set of genes contains a larger number of sociality genes than the background (26% vs 12%), and hence does not reflect balancing selection. A previous study also suggested that cheater genes experience balancing selection^6^, whereas we find that they show no differences in their signatures of selection compared with their background. The cause of the differences between the studies is unknown, but likely reflects several factors. First, although we focused on patterns across the CDS of these genes, the prior results were based primarily on variously sized genomic blocks (5, 10, or 20 kb) around the genes (which would typically contain multiple genes each). Second, the statistical evidence supporting the conclusions does not appear to have been subjected to correction for multiple testing, which is potentially problematic as many reported *p* values are very close to the significance threshold and many tests were performed at multiple genomic scales, making it difficult to assess some of the statistical support.

Although we have focused on the impact of direct selection shaping variation and divergence at social genes, other analyses of how social interactions impact molecular evolution in systems including *D. discoideum*^7^ have considered the impact of kin selection^13,36,37^. Kin selection (rather than direct selection) can result in the Red King process because it relies on the probabilistic association between individuals’ genotypes and their fitness^38^. Indeed, such dilution of selection has been shown to lead to elevated polymorphism at maternal effect genes^39,40^ (compared with homologs that are expressed in all individuals), which are thought to evolve by kin selection^36,41^, and at genes in a number of social insects^36,37^. In the case of *D. discoideum*, the impact of kin selection could potentially result in weaker selection on prestalk genes (which are altruistically sacrificed, and hence do not experience direct selection) compared with prespore genes (which presumably experience direct selection)^7^. This potential for kin selection has been contrasted with the potential impact of conditional selection, where prestalk genes might show strong signatures of conditional selection given that most cells become spores (ca. 70–80%), and hence selection on prestalk genes might be diluted proportionally. However, previous analyses of genes showing biased expression in prestalk and prespore cells^7^, as well as the results presented here (Supplementary Table 5, 13, 14, 16, 18, 19), show that the levels of polymorphism in the two sets of genes are not significantly different from 1:1, and hence do not support a role for either kin or conditional selection. This finding is perhaps unsurprising given that these two sets of genes are expressed across many other contexts (including the fact that most of these genes are actually expressed in both cell types, with ~90% showing at least 10% of their expression in the other cell type). Consequently, the impact of differences in expression between prestalk and prespore cells would be diminished by episodes of selection in other contexts. Together these findings provide little evidence in support of either kin selection, or differences in the strength of direct selection between prestalk and prespore biased genes.

A key challenge for studies aimed at understanding the molecular evolution of social genes is in first identifying representative social genes^4,42,43^. To provide broad and generalizable results, we used four different approaches to identify putative sets of social genes. Remarkably, we see relatively little overlap between these sets of genes, with no gene being identified by all four methods (Table 1). These sets of genes differ in their evolutionary signatures, but all follow the predictions of the Red King process, where variation in patterns of polymorphism (Fig. 4a–c) and divergence (Fig. 4d–f) can be predicted from the proportion of genes in each class that are conditionally expressed only in social stages.

Our ability to characterize signatures of selection and identify the importance of the Red King process was facilitated by the recognition that variation at synonymous sites is under selection. Because many molecular evolution studies, including previous analyses of social genes in *D. discoideum*^6,7^, are based on the a priori assumption that synonymous substitutions are neutral, they are potentially liable to misinterpret patterns of polymorphism and divergence. Synonymous sites are unlikely to be sources of functional (and potentially adaptive) variation, such as that underlying different social strategies (which presumably arise primarily from nonsynonymous differences). Therefore, elevated synonymous polymorphism is unlikely to be maintained by balancing selection and more likely reflects inefficient purifying selection on codon use. Thus, the fact that we typically see differences among groups of genes in their evolutionary signatures (for both polymorphism and divergence) at both synonymous and nonsynonymous sites suggests the same phenomenon is affecting the strength of selection at all sites in the CDS. Thus, despite the fact that previous studies of variation at social genes in this species have suggested that social genes show signatures consistent with patterns driven by social interactions^6,7^, we see no evidence in support of this conclusion.

In summary, we find that the Red King process, wherein the relative use and disuse of social genes across generations modulates the relative strength of selection they experience, provides a unifying explanation for large-scale evolutionary patterns. This conclusion does not rule out a role for other evolutionary processes, like the Red Queen, at some genes, but the impact is likely restricted to a relatively small collection of genes or sites. In the context of social conflict, the overall pattern of purifying selection at genes associated with the social stage (regardless of how they were identified) is consistent with there being an overall optimum, as expected under an ESS, but that selection is diluted owing to conditionality. Given that phenotypic studies have identified conspicuous differences between naturally occurring strains in all traits measured^17–19^, our results suggest that the observed diversity potentially reflects the inefficiency of selection to remove variation, rather than selection maintaining a diversity of alternative strategies.

## Methods

### Genomic DNA sequencing

Genomic DNA was extracted and sequenced from 58 *D. discoideum* strains and one divergent Mexican *Dictyostelium* strain (OT3A, which is characterized as *D. discoideum*, but could represent a close congener), all obtained from the Dicty Stock Center^44^. For DNA extraction, 10^9^ cells were collected after growth on nutrient media that contained *Klebsiella aerogenes*. Cells were re-suspended in KK2 and washed at least three times by centrifugation at 2200 rpm for 2 min to remove remaining bacteria. Nuclei were extracted from the pellet containing amoeba, followed by genomic DNA extraction as described elsewhere^29^. gDNA quality was assessed by agarose gel electrophoresis and a NanoDrop spectrophotometer (Thermo scientific). gDNA was quantified using a Qubit® fluorometer (Thermo scientific) before genomic libraries were prepared using Illumina TruSeq kit. Paired-end sequencing for reads ranging from 75–100 bp were obtained on an Illumina HiSeq sequencer. A second round of library sequencing was performed for strains NC105.1, DD185, K10, S109, QS102, NC85.2, and NC60.1 in order to increase the number of reads. To complement our de novo sequencing we also downloaded raw reads from NCBI Sequence Read Archive (SRP071575) of published genome sequence data from another 20 *D. discoideum* natural strains^29^ (Supplementary Table 20).

### Mapping and SNP calling

Reads were cleaned for adapters and quality trimmed using Trimmomatic^45^ allowing maximally two mismatches in seed alignments and extending and clipping if a score of 30 is reached. Leading and trailing bases with a quality <3 were removed, before scanning the reads with a 4-base sliding window and cutting if the average quality per base dropped below 15. Reads with a length of <36 bases after this process were then dropped. In order to separate *D. discoideum* reads from those derived from possible contaminants, trimmed reads were binned by simultaneously mapping them to the reference genome of *D. discoideum*, *Paraburkholderia xenovorans lb400*, *Burkholderia ubonensis*, *Paraburkholderia fungorum*, and *K. pneumoniae*; and assigning them according to the best mapping score using BBSplit, part of the BBMap package^46^. Genomes from the aforementioned bacterial species were downloaded from Ensembl Bacteria database^47^. Reads binned with *D. discoideum* or not mapped in the previous step where pooled together and mapped to the *D. discoideum* reference genome using NextGenMap^48^.

SNP calling was performed by comparison with the reference genome^32^ using the Genome Analysis Toolkit GATK^49^, following Best Practices recommendations for standard hard filtering parameters^50,51^. In brief, alignments were sorted and PCR duplicates marked using Picard tools^52^. Base quality score recalibration (BQSR) was performed by calling SNPs in each strain, filtering out sites with a Quality lower than 30, depth of coverage lower than 2, quality by depth (QD) <2, Fisher strand bias (FS) over 60 or Mean Mapping Quality (MQ) <40. Remaining SNPs were then used to perform BQSR using GATK. Variants were then jointly called on the 79 strains using GATK HaplotypeCaller and GenotypeGVCFs functions. Resulting SNPs were filtered with a static threshold of QD < 2.0 || FS > 60.0 || MQ < 30.0. As to maximize the number of informative sites for posterior analysis, whereas reducing the amount of noise introduced by missing genotypes in strains with low genome coverage or high diversity, we removed any strain with a missing call rate higher than 0.3, any site called in <90% of the remaining strains (i.e., in < 60 out of 67 strains), as well as any multiallelic site or indel. This results in a data set of 279,807 SNPs across 67 strains.

### Intraspecific evolutionary statistics

Parameters of genetic diversity (number of SNPs and the average nucleotide diversity, *π*) and Tajima’s *D* were estimated for genes with an average mapping of >50%, using the R package PopGenome^53^. The two diversity measures were estimated for coding regions, nonsynonymous and synonymous sites, and then scaled to the relevant mapped CDS length to obtain per site measures (where 78% of mapped sites on average are nonsynonymous and 22% synonymous, so total mapped CDS length was scaled accordingly when calculating per site measures for nonsynonymous and synonymous sites). Characterization of SNPs that introduce premature stop codons was performed by using SnpEff^54^. Genes with an average mapping ≤50% were considered to hold a PAV and were analyzed separately to assess if this type of structural genetic variation was more frequent among any group of social genes.

### Interspecific divergence

SNPs were further characterized as nonsynonymous (*n*) or synonymous (*s*) and segregating (*P*) or fixed (*D*) differences by comparison with a Mexican *Dictyostelium* isolate OT3A. Although this strain is annotated as *D. discoideum* in dictyBase^44^, the low mapping rate of our sequencing reads and the high divergence of this strain with respect to all other isolates suggest that this strain belongs to a different species, or at the very least, is an outgroup to the strains used in this study. We used this information to perform the McDonald–Kreitman test^33^ using the R Package PopGenome^53^. These counts were also included in the calculation of the Direction of Selection (*DoS*) statistic^34^. In both cases, the analysis was conducted for each gene individually, not by pooling all SNPs from genes pertaining to the same group.

To calculate rates of protein evolution we compared the reference genome of *D. discoideum*^32^ to OT3A. We first built the pseudo genome of OT3A by inserting SNPs for this strain (in comparison with the reference genome) into the reference genome, by using VCFtools software package^55^. CDSs for all genes from both genomes were extracted using gffread^56^ and rates of nonsynonymous (*K*_*a*_) and synonymous substitutions (*K*_*s*_) were estimated using R package seqinR^57^. The rate of protein evolution *K*_*a*_/*K*_*s*_ was calculated for each CDS and averaged for alternative transcripts of the same gene.

### Identification of sociality genes

To identify genes biased to the social (developmental) cycle of *D. discoideum*, we used data from several published RNA-seq experiments sampled from vegetative growth^21–23^ and from the developmental transcriptome^21^. In total, we used data from seven vegetative conditions (15 replicates) and 18 developmental time points during the social stage sampled at every 1–2 h (from hour 1–24, two replicates each)^21^. Reads were downloaded from NCBI Gene expression Omnibus (GEO: GSE61914), trimmed with skewer package^58^ and filtered for a minimum length of 20 bp and a mean Phred Quality score of 20. Remaining reads were pseudo-aligned to transcripts of the *D. discoideum* reference genome^32^ downloaded from Ensembl Protists database release 36^47^ and further quantified using Kallisto^59^. One hundred bootstrap samples were generated for each replicate to compute uncertainty estimates for the expression levels. Normalization was performed using the TMM method^60^ implemented in edgeR^61^ and scaled to coding sequence length, after discarding genes with less than two reads in less than two libraries.

Our analyses of differential expression across time points (Supplementary Figure 3) agreed with previous findings that genes upregulated 1 hour following starvation have GO categories consistent with a shift to multicellular social development^21^. We also find that the *Tgr* genes, which are known to play an important role in social interactions^29,62^ are upregulated at this stage (Supplementary Figure 4). Consequently, we considered the social stage to begin at the first hour. Therefore, data from hour 1–24 are considered to be part of the social library, whereas data hour zero and from all vegetative conditions are considered to be part of the vegetative libraries.

In order to define sociality genes, we averaged values for replicates and calculated an ISE, defined as the proportion of the total expression that appears in the social libraries^24^:

1 $$ISE = \frac{{\overline {{\mathrm{Social}}\;{\mathrm{libraries}}} }}{{\overline {{\mathrm{Vegetative}}\;{\mathrm{libraries}}} + \overline {{\mathrm{Social}}\;{\mathrm{libraries}}} }}$$

Sociality genes were defined as those with an index higher than 0.9.

We subjected the sociality genes to two increasingly stringent tests to account for any potential variation in expression profiles or leaky expression. In the first we considered a restricted subset of conserved developmentally expressed genes shared between at least two of the four clades of social amoebae that were identified by Schilde et al.^30^. In the second subset, we only considered genes with an expression level above a threshold that removed the lowest (bottom quartile) expressed genes (which are the ones most likely to reflect leaky expression patterns).

For the analysis of conserved genes, Schilde et al.^30^ used three different methods to identify developmentally biased genes conserved across species, which we combine into a single set of 852 genes (note that the analysis of Schilde et al. identified 856 genes, but one was duplicated in the list and three were removed because they are no longer recognized as gene models in the current version of the genome—Ensembl 2.7). This provides us with the largest set of genes and avoids potential biases introduced by any one of their methods. Although the Schilde genes were identified because of their association with development, we find that six genes had no detectable expression in vegetative or developmental libraries, and only 422 of the remaining 846 genes are sociality genes (i.e., have expression restricted to development). The analyses of these genes followed the same methods as we used for the full set of sociality genes, but carried out in the specific subset of genes in each of these two cases.

We tested whether any of the evolutionary parameters that differ between sociality and background genes are correlated to the ISE. The distribution of ISE values is strongly bi-modal, with a clear tranche of genes with values above 0.90 that we have assigned to the class of sociality genes (Supplementary Figure 2A). Because our comparison of sociality genes to their respective background has already assessed evolutionary signatures at the group of high ISE genes, the inclusion of that group in an analysis of ISE values is not only redundant, but likely to produce significant associations solely because of the categorical difference associated with the sociality genes. Therefore, for the analysis of ISE values we exclude sociality genes to ask whether the non-conditionally expressed genes show a relationship between the bias towards or away from sociality (measured by ISE) and their evolutionary signatures. We measured the correlation between ISE and each of the relevant evolutionary parameters and used a random permutation approach to assess the significance of correlations. For each permutation (from a total of 10,000), ISE values were held fixed, whereas the evolutionary parameters were randomly permuted (one at a time), generating the null distribution of correlations. *P* values were later corrected for multiple comparisons by applying the method *FDR*.

### Identification of chimerism genes

To identify genes showing differential expression under chimeric conditions we experimentally created clonal and all pairwise chimeric aggregations using three strains originating from the same geographical location (NC34.2, NC57.1, and NC87.1). Cells of each strain were grown in association with *K. aerogenes*, before washing by centrifugation in KK2 buffer. Washed cells were then plated on non-nutrient L28 purified agar (agar) at a density of 3.5 x 10^5 ^cells/cm^2^. For chimeric combinations we mixed equal numbers of cells from each strain. Aggregations were harvested after fourteen hours of development, when slugs had formed. This stage was chosen because previous work has demonstrated that the effects of chimeric development can be seen at this stage^29,63–65^. Development of each clone and chimeric combination was carried out in duplicate. For each replicate, slugs from 10 agar plates were pooled for RNA extraction using Trizol. RNA pools were sequenced on an Illumina TruSeq with 100 bp paired-end reads following standard protocols. This yielded between ~10^7^ and 2 × 10^7^ (mean ~1.5 × 10^7^) reads per RNA pool.

Preprocessing and mapping was performed as described above for the identification of sociality genes. In brief, reads were trimmed and filtered using the skewer package^58^ (min. length of 20 bp, mean Quality score of 20). They were then pseudo-aligned to *D. discoideum* transcripts^32^ obtained from Ensembl Protists database release 36^47^ and quantified using Kallisto^59^. One hundred bootstrap samples were generated for each replicate to compute uncertainty estimates for the expression levels. Genes with fewer than five reads in at least 47% of the libraries were discarded. Estimates of expression were then summarized to gene level and Wald test for differential expression was performed for chimeras and clonal samples by using sleuth^66^. Chimerism genes are then defined as those that are significantly upregulated in chimeric slugs (*FDR* adjusted *p* value < 0.05).

### Identification of chimerism genes

A list of 903 prespore and 998 prestalk genes was obtained from ref. ^7^, which is derived from an RNA-seq experiment that identified genes differentially expressed in these two cell subtypes^23^. For evolutionary analyses, these genes were compared against all genes in the expression data provided in ref. ^23^. One prespore and four prestalk genes in the prespore/prestalk list were not present in the original data, but were included in our analysis in both: the background and the specific groups of genes.

For the analysis of the impact of Red King processes on polymorphism, we included two extra sets of antagonism genes based on their expression bias between prestalk and prespore regions of the slug (corresponding to biases of ≥ 0.8 and ≥ 0.9 in either cell type). For this, we combined the data from ref. ^23^ with an additional set of data, which was generated as follows. *D. discoideum* cells transformed with either ecmAO-RFP or pspA-RFP reporter genes^67^ were developed to the slug stage. Slugs were collected in dissociation buffer (KK2, 10 mm EDTA) and dissociated through a G21 needle. Cells were re-suspended at 10^8^ cells/ml and cell clumps removed by filtration. RFP expressing cells were purified using a BD FACSaria flow sorter. Total RNA was extracted using TRI Reagent, before rRNA depletion using Ribminus^TM^ Eukaryotic kit (Invitrogen). In total, 200–500 ng of rRNA depleted RNA was reverse transcribed, fragmented and size selected for 150–250 bp cDNA fragments. cDNA was amplified using strand specific primers and sequenced using a SOLiD 4 system. Expression biases were calculated separately for each dataset as the proportion of the total expression that appears in the prestalk samples compared with prespore samples, and vice versa. Biological replicates were averaged and expression bias was then calculated as follows:

2 $${\mathrm{Prestalk}}\;{\mathrm{bias}} = \frac{{{\mathrm{Prestalk}}\;{\mathrm{expression}}}}{{{\mathrm{Prestalk}}\;{\mathrm{expression}} + {\mathrm{Prespore}}\;{\mathrm{expression}}}}$$

3 $${\mathrm{Prespore}}\;{\mathrm{bias}} = \frac{{{\mathrm{Prespore}}\;{\mathrm{expression}}}}{{{\mathrm{Prestalk}}\;{\mathrm{expression}} + {\mathrm{Prespore}}\;{\mathrm{expression}}}}$$

Genes were included in each set if the average of the two indices was larger than the given threshold (e.g., if the average of the bias calculated from both sets was ≥ 0.8 the gene would have been included in the 0.8 bias set).

### Identification of cheater genes

Previous work has identified mutations that result in a facultative reduction of cooperative behavior when *D. discoideum* strains grown in chimeras with a different strain^28^. These genes were identified by screening of insertional mutagenesis libraries. A fraction of these mutations, occurring in intergenic regions, were discarded. The remaining mutations affect a total of 99 genes, which we referred to as cheater genes.

### Conditionally expressed non-social genes

We identified genes with presumed conditional expression outside of the social phase as those that were not expressed in any of the mRNA libraries used in our analysis of sociality genes. These conditional genes are presumably expressed under some conditions, but are not expressed under any of the conditions included in the data sets we analyzed. We compared these genes to the non-conditional genes (i.e., genes with non-zero expression across RNA-seq experiments with sociality genes removed) following the same methods we used to compare other gene classes to their relevant background set.

### GO enrichment

GO terms for biological process, cellular component, and molecular processes were obtained from Dictybase^44^. Enrichment analyses of GO categories in sociality, chimerism, antagonism, and cheater genes were performed in R and statistical significance was assessed after *FDR* adjustment of one tail *p* values from Monte Carlo sampling (see below).

### Analyses and significance testing

Data manipulation and all analyses of categorical variables were performed in R version 3.3.0 and RStudio version 0.99.902, using built-in functions. For all continuous variables, expected values were estimated using a linear model fitted using the mixed procedure in SAS 9.4. In the linear models used to analyze polymorphism and divergence we included mapped CDS length (linear and quadratic terms) and maximum expression level (linear and quadratic terms) to account for variation associated with differences in gene length (which can bias measures based on counting of SNPs) and gene expression levels. Because estimates of evolutionary statistics improve with gene length (and can be downwardly biased for short genes), we removed genes in the bottom quartile of gene lengths when analysing all continuous variables. The inclusion of these covariates and the removal of the shortest quartile of genes do not alter the overall qualitative results.

Unless otherwise explicitly stated in the text, significance was assessed by randomization (i.e., permutation) tests. For each evolutionary analysis, 10,000 samples (*s*) of the same size of the group of genes being tested (sociality, chimerism, antagonism, or cheater genes) were taken. Each sample was either used for the fitting of the linear model (for continuous variables) or the number of genes showing a particular feature was computed (for categorical variables)—both cases resulting in a distribution of 10,000 random samples. Expected values were calculated as the overall mean of this random distribution. Significance of categorical variables were first assessed by comparing observed values to the 95% confidence interval (CI). When these values lie outside the CI, numeric two tail *p* values were calculated as twice the number of times that the observed count for the particular group of genes did not exceed the one in the randomly generated subset divided by 10,000. Two tail *p* values for continuous variables were obtained similarly, but using averages values instead of counts. For every statistical test, an *FDR* (Benjamini–Hochberg) correction for multiple tests was performed.

### Reporting summary

Further information on research design is available in the Nature Research Reporting Summary linked to this article.

## Supplementary information

Supplementary Information

Reporting Summary

Source Data

## Data Availability

All data generated or used in the current study are publicly available and all relevant variables used in all analyses are available in the Source Data file. The list of genetic variants used in all analyses are available from the EMBL-EBI European Variation Archive (EVA) (project: PRJEB28260 and analysis: ERZ681043). The transcriptome data used in the analysis of sociality genes were downloaded from NCBI Gene expression Omnibus (GEO: GSE61914). The list of prespore and prestalk genes used in the analysis of antagonism genes was obtained from ref. ^7^, which was combined with a list of all genes included in the original RNA-seq experiment from ref. ^23^. The list of cheater genes is available from ref. ^28^. These gene lists are also included in the Source Data file. The RNA-seq (transcriptome) data sets from the comparison of clonal and chimeric slugs (used in the analysis of conflict genes) are available from the NCBI Gene Expression Omnibus (GSE118081). The RNA-seq (transcriptome) data from the comparison of prestalk and prespore regions (used to identify genes with biased expression in these regions for the linear model testing the effect of proportion of sociality genes) are available from the NCBI Sequence Read Archive (SRA) (accession: PRJNA543665). The data underlying Figures. 1–4 are available in the Source Data file.
