# Supplementary material for: Conditional expression explains molecular evolution of social genes in a microbe
Source: Nat Commun. 2019 Jul 23;10:3284. doi: 10.1038/s41467-019-11237-2 (PMC6650454; doi:10.1038/s41467-019-11237-2)
Supplement: Supplementary file 1 — Supplementary Information [file 41467_2019_11237_MOESM1_ESM.pdf]

Supplementary Information

Conditional expression explains molecular evolution of social genes in a microbe

Janaina Lima de Oliveira, Atahualpa Castillo Morales et al.

Contents:

1. **Supplementary Figure 1.** *D. discoideum* life cycle and identification of different groups of social genes.

2. **Supplementary Figure 2.** Identification and characterization of sociality genes.

3. **Supplementary Figure 3.** Sliding widow analysis of differential expression.

4. **Supplementary Figure 4.** Differential expression of *Tgr* genes through development.

5. **Supplementary Table 1.** GO enrichment analysis for sociality genes.

6. **Supplementary Table 2.** GO enrichment analysis for chimerism genes.

7. **Supplementary Table 3.** GO enrichment analysis for antagonism genes.

8. **Supplementary Table 4.** GO enrichment analysis for cheater genes.

9. **Supplementary Table 5.** Average number of SNPs (SNP/site) for social genes.

10. **Supplementary Table 6.** Comparison of observed and expected evolutionary parameters for sociality and antagonism genes based on the presence of each class in the other.

11. **Supplementary Table 7.** Comparison of observed and expected evolutionary parameters for antagonism genes with all sociality genes removed from the set.

12. **Supplementary Table 8.** Comparison of observed and expected evolutionary parameters for sociality genes with all antagonism genes removed from the set.

13. **Supplementary Table 9.** Comparison of evolutionary parameters at the subset of sociality genes relative to the background of all of the genes identified by Schilde et al.

14. **Supplementary Table 10.** Comparison of evolutionary parameters for sociality genes compared to the relevant background set of genes in a subset of genes that excludes those in the bottom quartile of expression.

15. **Supplementary Table 11.** Correlations between the index of social expression (ISE) and the set of evolutionary parameters that differ between sociality and the background set of genes.

16. **Supplementary Table 12.** Comparison of evolutionary parameters for conditional genes (observed) compared to those expected.

17. **Supplementayr Table 13.** Complementary neutrality tests for social genes. Fu & Li's statistics compare external and internal branches of a genealogical tree.

18. **Supplementary Table 14.** Enrichment analysis of social genes evolving under balancing selection as defined by different cutoffs of Tajima's *D*.

19. **Supplementary Table 15.** Intraspecific variation in sociality genes excluding 13 genes evolving under balancing selection.

20. **Supplementary Table 16.** Enrichment analysis of social genes showing strong signatures of selection.

21. **Supplementary Table 17.** Comparison of evolutionary parameters at genes showing biased expression in either prestalk and prespore cells relative to the background of all of the genes expressed in the same cells.

22. **Supplementary Table 18.** Evolutionary statistics for prespore and prestalk genes.

23. **Supplementary Table 19.** Enrichment analysis of the number of prespore and prestalk genes carrying at least one mutation that introduces a stop codon or results in a partial deletion (presence/absence variation).

24. **Supplementary Table 20.** Genome sequencing statistics for the strains included in analyses.

25. **Supplementary References.**

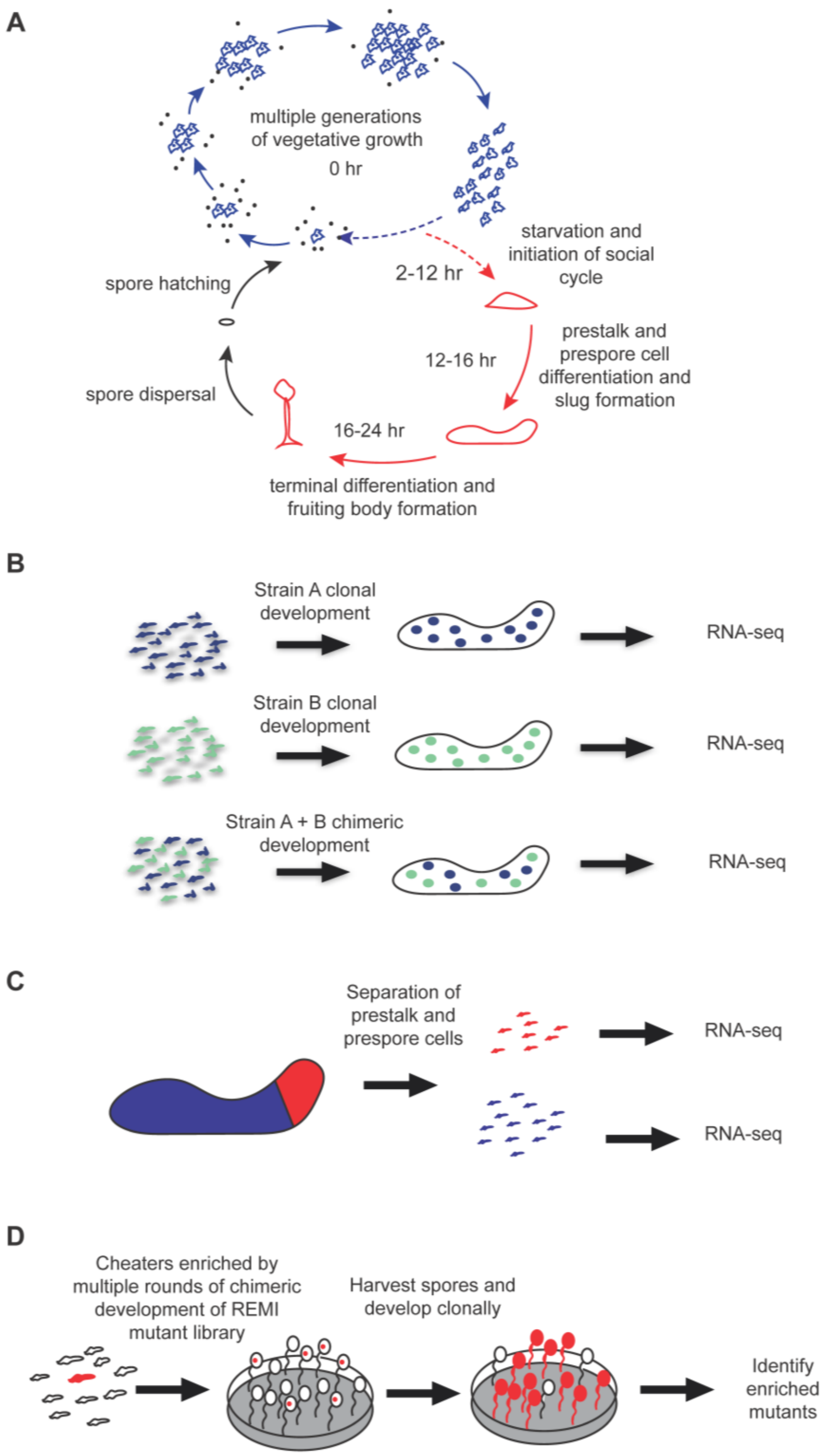

Supplementary Figure 1. *D. discoideum* life cycle and identification of different groups of social genes. **A)** Schematic figure showing the *D. discoideum* life cycle. Cells can go through many generations of vegetative life cycle (blue) whilst bacterial food is available. Multicellular social development (red) is a conditional strategy that is only entered if food supplies are depleted (dotted red arrow). The index of social expression was calculated by comparing transcript abundance at 0hr to all social stages (2-24 hr) using data from ref. 1. **B)** Chimerism genes were identified by comparing the transcriptome of strains that went through the social (developmental) stage clonally to those same strains mixed in chimeric development (where the green and blue cells are meant to represent different genotypes). **C)** Antagonism genes were identified comparing the transcriptome of isolated prestalk (red) and prespore (blue) cell populations[2,3]. **D)** Cheater genes were identified in a REMI screen designed to enrich for mutants (red, with all others appearing as white) that are overrepresented in the spore cell population after chimeric development[4].

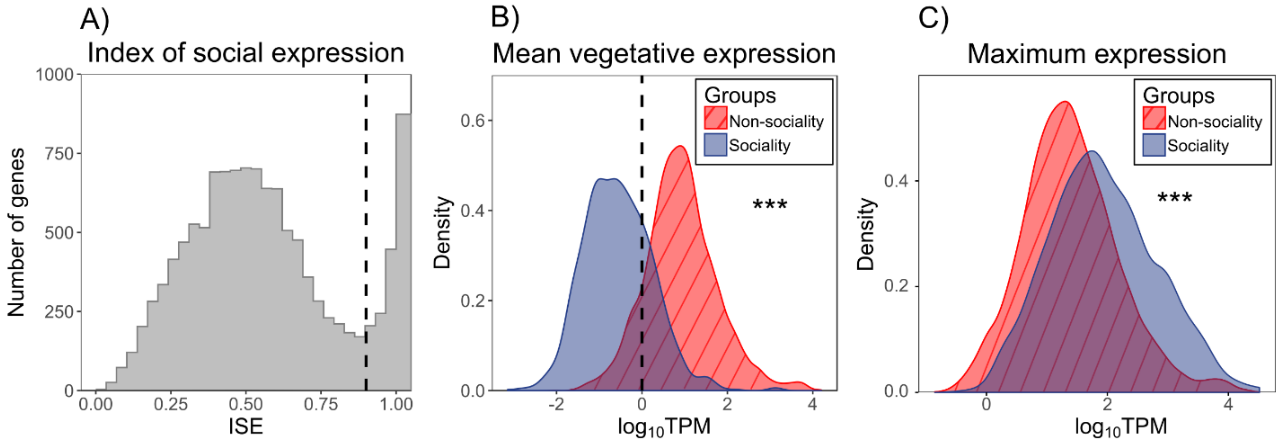

Supplementary Figure 2. Identification and characterization of sociality genes. **A)** Distribution of values for the Index of Social Expression (ISE). The dashed line represents the cutoff of ISE = 0.9 used to define sociality (ISE > 0.9) and non-sociality (ISE ≤ 0.9) genes. **B)** Sociality genes have little or no expression during vegetative growth (Kolmogorov-Smirnov test: $p < 10^{-15}$), suggesting that they are conditional to the social stage. **C)** Although conditional to a fraction of generations, sociality genes are usually required at high levels when expressed (Kolmogorov-Smirnov test: $p < 10^{-15}$). Note that the x-axis ranges differ in parts B and C, which reflects differences in the properties of mean versus maximum expression.

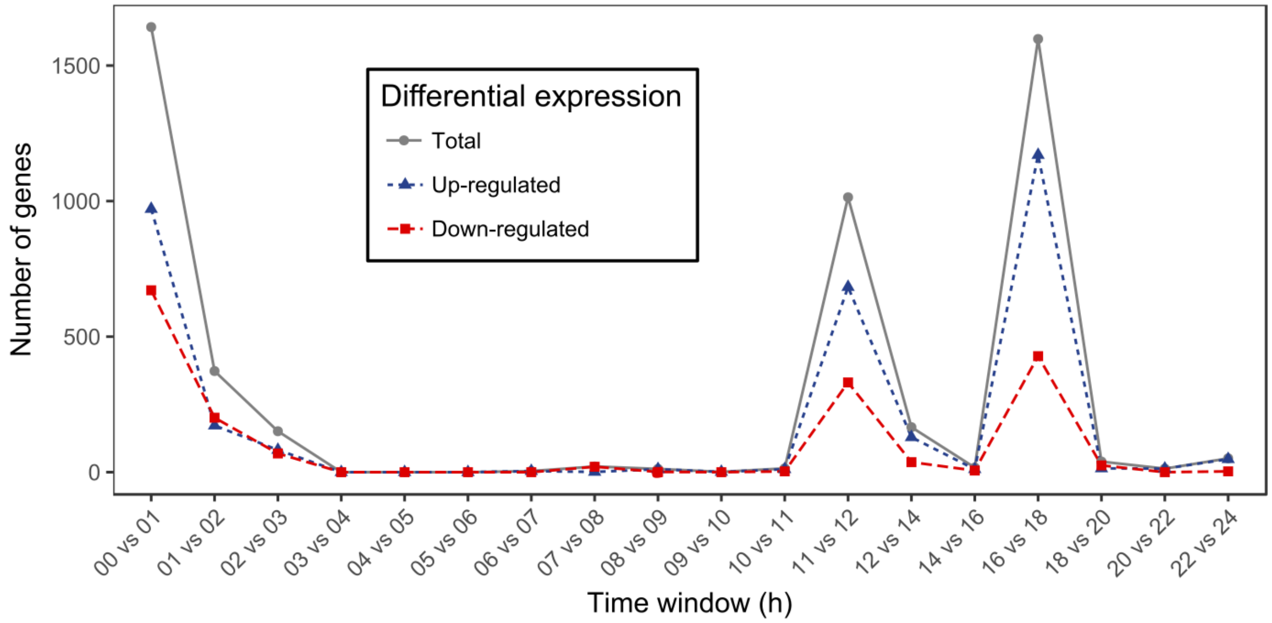

Supplementary Figure 3. Sliding widow analysis of differential expression. By computing the number of differentially expressed genes between a given time point and the subsequent one (*t* versus *t+1*), an analysis of the developmental transcriptome reveals three major points of global changes in expression patterns. The first step marks the beginning of development (00-01h), suggesting that conditional expression of developmental genes is observed as early as within the first hour of starvation. The second and third peaks are related to switches from loose aggregates to multicellularity (11-12h) and beginning of culmination (16-18h), respectively (see ref. 1). Note that time windows progress in one-hour intervals until hour 12, after which they progress in two-hour intervals through to hour 24.

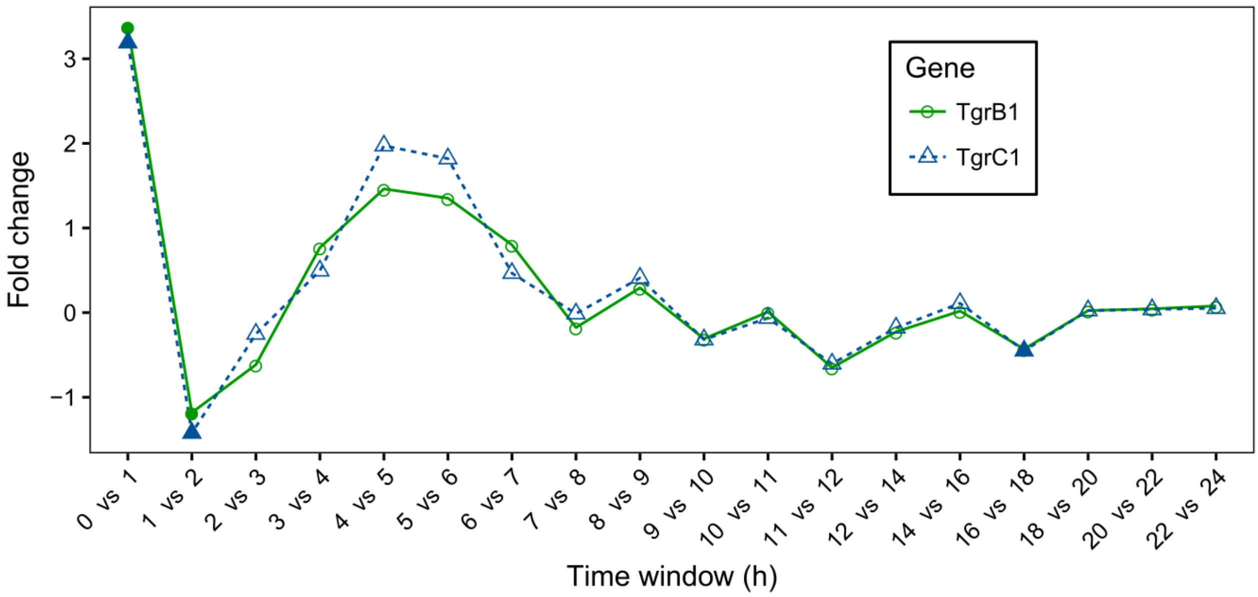

Supplementary Figure 4. Differential expression of *Tgr* genes through development. The pair of developmental genes *TgrB1* and *TgrC1* is up-regulated (filled symbols, positive fold change) on the onset of development, between the vegetative stage and the first hour of starvation. They are further down-regulated between hours 1 and 2, and again at the beginning of culmination (hours 16 and 18) (filled symbols, negative fold change). In other time points, transcripts of these genes are accumulated and increase levels, but are not differentially expressed (empty symbols).

Supplementary Table 1. GO enrichment analysis for sociality genes. We used a randomization procedure to test whether this group of genes is enriched for GO terms of biological process, cellular component and molecular function. For each GO term, we generated a set of 10,000 random groups of size *N* (where *N* is the number of sociality genes) sampled from a set that contains sociality genes and its corresponding background set of genes. In each randomization we computed the number of genes associated to the GO term being tested and used the distribution of the counts across randomizations to calculate the one-tail p-values. Only terms overrepresented among sociality genes after *FDR* correction are shown.

GOID	GO Term	Obs	Exp	*P*	FDR *p*
	**Biological Process**				
GO:0030198	extracellular matrix organization	29	4.6	$<10^{-4}$	$<10^{-4}$
GO:0030435	sporulation resulting in formation of a cellular spore	24	10.26	$<10^{-4}$	$<10^{-4}$
GO:0031154	culmination involved in sorocarp development	28	12.71	$<10^{-4}$	$<10^{-4}$
GO:1902168	response to catechin	7	1.38	$<10^{-4}$	$<10^{-4}$
GO:0008150	biological_process	411	263.12	$<10^{-4}$	$<10^{-4}$
GO:000NABP	no biological process annotation	803	695.64	$<10^{-4}$	$<10^{-4}$
	**Cellular Component**				
GO:0005576	extracellular region	83	26.82	$<10^{-4}$	$<10^{-4}$
GO:0016021	integral component of membrane	369	308.21	$<10^{-4}$	$<10^{-4}$
GO:0031012	extracellular matrix	30	4.84	$<10^{-4}$	$<10^{-4}$
GO:0005575	cellular_component	438	318.09	$<10^{-4}$	$<10^{-4}$
GO:000NACC	no cellular component annotation	702	630.71	0.0002	0.0264
	**Molecular Function**				
GO:0004497	monooxygenase activity	27	8.62	$<10^{-4}$	$<10^{-4}$
GO:0004553	hydrolase activity, hydrolyzing O-glycosyl compounds	25	9.18	$<10^{-4}$	$<10^{-4}$
GO:0005201	extracellular matrix structural constituent	27	4.41	$<10^{-4}$	$<10^{-4}$
GO:0005506	iron ion binding	23	9.73	$<10^{-4}$	$<10^{-4}$
GO:0016705	oxidoreductase activity, acting on paired donors, with incorporation or reduction of molecular oxygen	21	6.96	$<10^{-4}$	$<10^{-4}$
GO:0020037	heme binding	26	10.02	$<10^{-4}$	$<10^{-4}$
GO:0030246	carbohydrate binding	43	14.42	$<10^{-4}$	$<10^{-4}$
GO:0001646	cAMP receptor activity	6	1.11	0.0001	0.0159
GO:0030248	cellulose binding	28	14.05	0.0003	0.0433
GO:0003674	molecular_function	370	268.910	$<10^{-4}$	$<10^{-4}$
GO:000NAMF	no molecular function annotation	786	669.990	$<10^{-4}$	$<10^{-4}$

Supplementary Table 2. GO enrichment analysis for chimerism genes. We used a randomization procedure to test whether this group of genes is enriched for GO terms of biological process, cellular component and molecular function. For each GO term, we generated a set of 10,000 random groups of size *N* (where *N* is the number of chimerism genes) sampled from a set that contains chimerism genes and its corresponding background set of genes. In each randomization we computed the number of genes associated to the GO term being tested and used the distribution of the counts across randomizations to calculate the one-tail *p*-values. Only terms overrepresented among chimerism genes after *FDR* correction are shown.

GOID	GO Term	Obs	Exp	*P*	FDR *p*
	**Biological Process**				
GO:0006096	glycolytic process	6	0.29	$<10^{-4}$	$<10^{-4}$
GO:0006099	tricarboxylic acid cycle	7	0.40	$<10^{-4}$	$<10^{-4}$
GO:0006108	malate metabolic process	4	0.10	$<10^{-4}$	$<10^{-4}$
GO:0006164	purine nucleotide biosynthetic process	5	0.18	$<10^{-4}$	$<10^{-4}$
GO:0006338	chromatin remodeling	4	0.28	$<10^{-4}$	$<10^{-4}$
GO:0006457	protein folding	12	1.21	$<10^{-4}$	$<10^{-4}$
GO:0006458	'de novo' protein folding	5	0.29	$<10^{-4}$	$<10^{-4}$
GO:0006471	protein ADP-ribosylation	3	0.09	$<10^{-4}$	$<10^{-4}$
GO:0006520	cellular amino acid metabolic process	5	0.18	$<10^{-4}$	$<10^{-4}$
GO:0006531	aspartate metabolic process	2	0.03	$<10^{-4}$	$<10^{-4}$
GO:0006532	aspartate biosynthetic process	3	0.05	$<10^{-4}$	$<10^{-4}$
GO:0006536	glutamate metabolic process	3	0.09	$<10^{-4}$	$<10^{-4}$
GO:0008152	metabolic process	24	5.67	$<10^{-4}$	$<10^{-4}$
GO:0009408	response to heat	4	0.15	$<10^{-4}$	$<10^{-4}$
GO:0031589	cell-substrate adhesion	5	0.46	$<10^{-4}$	$<10^{-4}$
GO:0046689	response to mercury ion	6	0.76	$<10^{-4}$	$<10^{-4}$
GO:0055114	oxidation-reduction process	22	6.70	$<10^{-4}$	$<10^{-4}$
GO:0061077	chaperone-mediated protein folding	5	0.44	$<10^{-4}$	$<10^{-4}$
GO:0000492	box C/D snoRNP assembly	2	0.03	0.0001	0.0092
GO:0006189	'de novo' IMP biosynthetic process	3	0.11	0.0001	0.0092
GO:0030435	sporulation resulting in formation of a cellular spore	7	1.18	0.0001	0.0092
GO:0070212	protein poly-ADP-ribosylation	2	0.03	0.0001	0.0092
GO:0008652	cellular amino acid biosynthetic process	4	0.32	0.0002	0.0168
GO:0019752	carboxylic acid metabolic process	3	0.17	0.0002	0.0168
GO:0006807	nitrogen compound metabolic process	3	0.18	0.0003	0.0216
GO:0010421	hydrogen peroxide-mediated programmed cell death	3	0.14	0.0003	0.0216
GO:0010918	positive regulation of mitochondrial membrane potential	3	0.14	0.0003	0.0216
GO:0019538	protein metabolic process	2	0.03	0.0003	0.0216
GO:0006094	gluconeogenesis	3	0.16	0.0004	0.0269
GO:0009617	response to bacterium	6	0.99	0.0004	0.0269
GO:0000398	mRNA splicing, via spliceosome	6	1.04	0.0005	0.0315
GO:0046956	positive phototaxis	3	0.17	0.0005	0.0315
GO:0006538	glutamate catabolic process	2	0.05	0.0007	0.0415

GO:0046847	filopodium assembly	3	0.21	0.0007	0.0415
GO:0006734	NADH metabolic process	2	0.05	0.0008	0.0448
GO:0051103	DNA ligation involved in DNA repair	3	0.17	0.0008	0.0448
GO:0006273	lagging strand elongation	3	0.19	0.0009	0.0491
	**Cellular Component**				
GO:0005634	Nucleus	33	16.65	$<10^{-4}$	$<10^{-4}$
GO:0005681	spliceosomal complex	6	0.94	$<10^{-4}$	$<10^{-4}$
GO:0005737	cytoplasm	61	19.66	$<10^{-4}$	$<10^{-4}$
GO:0005739	mitochondrion	19	6.13	$<10^{-4}$	$<10^{-4}$
GO:0005759	mitochondrial matrix	7	1.05	$<10^{-4}$	$<10^{-4}$
GO:0005829	Cytosol	21	5.27	$<10^{-4}$	$<10^{-4}$
GO:0045335	phagocytic vesicle	34	4.87	$<10^{-4}$	$<10^{-4}$
GO:0097255	R2TP complex	2	0.03	$<10^{-4}$	$<10^{-4}$
GO:0005832	chaperonin-containing T-complex	5	0.21	0.0001	0.0073
GO:0000812	Swr1 complex	2	0.05	0.0004	0.0264
GO:0008540	proteasome regulatory particle, base subcomplex	3	0.17	0.0006	0.0330
GO:0044613	nuclear pore central transport channel	2	0.05	0.0006	0.0330
	**Molecular Function**				
GO:0000166	nucleotide binding	41	16.93	$<10^{-4}$	$<10^{-4}$
GO:0003824	catalytic activity	32	6.88	$<10^{-4}$	$<10^{-4}$
GO:0004069	L-aspartate:2-oxoglutarate aminotransferase activity	2	0.03	$<10^{-4}$	$<10^{-4}$
GO:0004352	glutamate dehydrogenase (NAD+) activity	2	0.03	$<10^{-4}$	$<10^{-4}$
GO:0005524	ATP binding	44	13.51	$<10^{-4}$	$<10^{-4}$
GO:0016491	oxidoreductase activity	22	6.78	$<10^{-4}$	$<10^{-4}$
GO:0016874	ligase activity	9	1.46	$<10^{-4}$	$<10^{-4}$
GO:0030170	pyridoxal phosphate binding	6	0.67	$<10^{-4}$	$<10^{-4}$
GO:0044183	protein binding involved in protein folding	5	0.31	$<10^{-4}$	$<10^{-4}$
GO:0051082	unfolded protein binding	12	0.81	$<10^{-4}$	$<10^{-4}$
GO:0031072	heat shock protein binding	3	0.08	0.0001	0.0144
GO:0004386	helicase activity	6	1.09	0.0003	0.0340
GO:0008483	transaminase activity	4	0.25	0.0003	0.0340
GO:0016620	oxidoreductase activity, acting on the aldehyde or oxo group of donors, NAD or NADP as acceptor	4	0.23	0.0003	0.0340

Supplementary Table 3. GO enrichment analysis for antagonism genes. We used a randomization procedure to test whether this group of genes is enriched for GO terms of biological process, cellular component and molecular function. For each GO term, we generated a set of 10,000 random groups of size *N* (where *N* is the number of antagonism genes) sampled from a set that contains antagonism genes and its corresponding background set of genes. In each randomization we computed the number of genes associated to the GO term being tested and used the distribution of the counts across randomizations to calculate the one-tail *p*-values. Only terms overrepresented among antagonism genes after *FDR* correction are shown.

GOID	GO Term	Obs	Exp	*P*	FDR *p*
	**Biological Process**				
GO:0008299	isoprenoid biosynthetic process	9	2.22	$<10^{-4}$	$<10^{-4}$
GO:0008150	biological_process	382	300.70	$<10^{-4}$	$<10^{-4}$
	**Cellular Component**				
GO:0005856	cytoskeleton	42	22.85	$<10^{-4}$	$<10^{-4}$
GO:0005938	cell cortex	34	16.96	$<10^{-4}$	$<10^{-4}$
GO:0016020	membrane	473	400.65	$<10^{-4}$	$<10^{-4}$
GO:0016021	integral component of membrane	427	352.00	$<10^{-4}$	$<10^{-4}$
GO:0005576	extracellular region	57	30.58	0.0001	0.0082
GO:0005615	extracellular space	87	59.78	0.0001	0.0082
GO:0042995	cell projection	12	3.97	0.0001	0.0082
GO:0005575	cellular_component	441	363.13	$<10^{-4}$	$<10^{-4}$
	**Molecular Function**				
GO:0003779	actin binding	42	19.60	$<10^{-4}$	$<10^{-4}$
GO:0005515	protein binding	67	42.59	$<10^{-4}$	$<10^{-4}$
GO:0003674	molecular_function	375	307.12	$<10^{-4}$	$<10^{-4}$

Supplementary Table 4. GO enrichment analysis for cheater genes. We used a randomization procedure to test whether this group of genes is enriched for GO terms of biological process, cellular component and molecular function. For each GO term, we generated a set of 10,000 random groups of size *N* (where *N* is the number of cheater genes) sampled from a set that contains cheater genes and its corresponding background set of genes. In each randomization we computed the number of genes associated to the GO term being tested and used the distribution of the counts across randomizations to calculate the one-tail *p*-values. Only terms overrepresented among cheater genes after *FDR* correction are shown.

GOID	GO Term	Obs	Exp	*P*	FDR *p*
	**Biological Process**				
GO:0035176	social behavior	24	0.23	$<10^{-4}$	$<10^{-4}$
	**Cellular Component**				
GO:0005575	cellular_component	35	17.55	$<10^{-4}$	$<10^{-4}$
	**Molecular Function**				
GO:0016301	kinase activity	11	2.58	$<10^{-4}$	$<10^{-4}$

Supplementary Table 5. Average number of SNPs (SNP/site) for social genes. Expected values and the respective two-tailed *p*-values were obtained from randomization distributions. For each group of social genes, we generated a set of 10,000 random groups of size *N* (where *N* is the number of genes in that particular group) sampled from a set that contains that group of social genes and its corresponding background set of genes (for the prespore and prestalk, the background for each is the combination of the two categories). Two-tailed *p*-values are defined as the probability of obtaining a mean as extreme as the observed only due to chance. Significant *p*-values after *FDR* correction for multiple tests are highlighted in bold (*FDR* < 0.05). FDR correction was done in two separate sets, one for the four main classes of genes and a second set that included these four plus the p-values from the prespore and prestalk analysis (since this is a nested analysis). Values smaller than 10^{-5} are listed as zeros.

Sites	Group	Expected ($x10^{-3}$)	Observed ($x10^{-3}$)	*p* (*FDR*)
CDS	Sociality	4.70	7.28	**<0.001**
	Chimerism	4.56	4.41	0.74
	Antagonism	4.70	5.15	**<0.001**
	Prespore	5.03	5.32	0.071
	Prestalk	5.02	4.75	0.071
	Presp-Prest	0	0.57	0.071
	Cheater	4.69	5.07	0.56
Nonsynonymous	Sociality	3.72	6.35	**<0.001**
	Chimerism	3.55	3.20	0.47
	Antagonism	3.73	4.18	**<0.001**
	Prespore	4.01	4.29	0.071
	Prestalk	4.01	3.74	0.071
	Presp-Prest	0	0.55	0.071
	Cheater	3.72	4.20	0.47
Synonymous	Sociality	8.10	10.52	**<0.001**
	Chimerism	8.08	8.66	0.47
	Antagonism	8.11	8.55	**0.034**
	Prespore	8.59	8.92	0.17
	Prestalk	8.59	8.26	0.17
	Presp-Prest	0	0.66	0.17
	Cheater	8.08	8.14	0.95

Supplementary Table 6. Comparison of observed and expected evolutionary parameters for sociality and antagonism genes based on the presence of each class in the other. Expected values and probabilities (*p*) are from 10,000 random permutations where genes were randomly sampled such that the final set had the same proportion of genes coming from the other class as that observed in the original data. For example, in the case of the sociality expected values, sets of antagonism and non-antagonism genes were sampled to create sets of 'sociality' genes contained the observed proportion of antagonism genes. For each permutation, means are expected values from a linear model that accounts for variation in expression and mapped CDS length (see Methods). Two-tailed *p*-values are defined as the probability of obtaining a mean as extreme as the observed only due to chance. Significant *p*-values after *FDR* correction for multiple tests are highlighted in bold (*FDR* < 0.05). *parameter values that have been multiplied by 10^3.

Parameter	Sociality (expected)	Sociality (observed)	*p* sociality	Antagonism (expected)	Antagonism (observed	*p* antagonism
π/site*	0.77	12.00	**<0.001**	0.79	0.83	0.45
π_a/site*	0.60	1.04	**<0.001**	0.63	0.65	0.52
π_s/site*	1.35	1.80	**<0.001**	1.28	1.30	0.17
SNP/site	4.81	7.28	**<0.001**	5.03	5.15	0.51
SNP/site	3.83	6.35	**<0.001**	4.07	4.18	0.46
SNP/site	8.21	10.52	**<0.001**	8.40	8.54	0.55
K_a/K_s	0.21	0.28	**<0.001**	0.21	0.21	0.84
K_a	1.01	1.53	**<0.001**	1.05	1.09	0.52
K_s	6.91	8.97	**<0.001**	7.05	7.15	0.64

Supplementary Table 7. Comparison of observed and expected evolutionary parameters for antagonism genes with all sociality genes removed from the set. Expected values and the respective two-tailed *p*-values were obtained from randomization distributions. For each randomization, we generated a set of 10,000 random groups of size *N* (where *N* is the number of genes in that particular group) sampled from a set that contained antagonism genes and their appropriate background set, but with all sociality genes removed. Two-tailed *p*-values are defined as the probability of obtaining a mean as extreme as the observed only due to chance. Significant *p*-values after *FDR* correction for multiple tests are highlighted in bold (*FDR* < 0.05). The observed and expected values for the full set of antagonism genes ('all') are shown for comparison. *parameter values have been multiplied by 10^3.

Parameter	Expected	Observed	*p* (FDR)	Expected (all)	Observed (all)
π/site*	0.70	0.72	0.91	0.75	0.82
π_a/site*	0.53	0.54	0.91	0.58	0.65
π_s/site*	1.27	1.34	0.73	1.32	1.44
SNP/site (CDS)*	4.33	4.32	0.91	4.70	5,15
SNP/site (nonsynon.)*	3.36	3.34	0.91	3.73	4.18
SNP/site (synon.)*	7.80	7.85	0.91	8.11	8.55
K_a/K_s	0.20	0.19	0.91	0.21	0.22
K_a*	0.94	0.95	0.91	0.99	1.09
K_s*	6.61	6.64	0.91	6.86	7.15

Supplementary Table 8. Comparison of observed and expected evolutionary parameters for sociality genes with all antagonism genes removed from the set. Expected values and the respective two-tailed *p*-values were obtained from randomization distributions. For each randomization, we generated a set of 10,000 random groups of size *N* (where *N* is the number of genes in that particular group) sampled from a set that contained sociality genes and their appropriate background set, but with all antagonism genes removed. Two-tailed *p*-values are defined as the probability of obtaining a mean as extreme as the observed only due to chance. Significant *p*-values after *FDR* correction for multiple tests are highlighted in bold (*FDR* < 0.05). The observed and expected values for the full set of sociality genes ('all') are shown for comparison. *parameter values have been multiplied by 10^3.

Parameter	Expected	Observed	*p* (FDR)	Expected (all)	Observed (all)
π/site*	0.71	1.18	**<0.001**	0.73	1.21
π_a/site*	0.56	1.02	**<0.001**	0.55	1.04
π_s/site*	1.27	1.80	**<0.001**	1.33	1.84
SNP/site (CDS)*	4.56	6.91	**<0.001**	4.70	7.28
SNP/site (nonsynon.)*	3.59	6.00	**<0.001**	3.72	6.35
SNP/site (synon.)*	8.00	10.14	**<0.001**	8.10	10.52
K_a/K_s	0.21	0.26	**0.022**	0.18	0.27
K_a*	0.97	1.47	**<0.001**	0.90	1.47
K_s*	6.66	8.88	**<0.001**	6.90	8.98

Supplementary Table 9. Comparison of evolutionary parameters at the subset of sociality genes [Obs (Schilde)] relative to the background of all of the genes identified by Schilde et al.[5] [Exp (Schilde)]. Two-tailed *p*-values are defined as the probability of obtaining a mean as extreme as the observed value due to chance. Significant *p*-values after *FDR* correction for multiple tests are highlighted in bold (*FDR* < 0.05). The observed values of each parameter measured in the full set of sociality genes [Obs (all)] and the value expected from the full set of genes [Exp (all)] are shown for comparison. *parameter values have been multiplied by 10^3.

Parameter	Exp (Schilde)	Obs (Schilde)	*p* (*FDR*)	Exp (all)	Obs (all)
π/site*	0.74	0.90	**<0.001**	0.73	1.21
π_a/site*	0.55	0.69	**<0.001**	0.55	1.04
π_s/site*	1.37	1.59	**0.002**	1.33	1.84
Tajima's *D*	−0.71	−0.70	0.82	−0.65	−0.71
K_a/K_s	0.16	0.17	0.70	0.18	0.27
K_a*	0.91	1.07	**0.005**	0.90	1.47
K_s*	8.10	9.76	**<0.001**	6.90	8.98
DoS	−0.044	−0.066	0.13	−0.022	−0.054
$D_n/(D_n + D_s)$	0.54	0.55	0.66	0.55	0.59
$P_n/(P_n + P_s)$	0.59	0.61	**0.005**	0.58	0.65

Supplementary Table 10. Comparison of evolutionary parameters for sociality genes (Obs) compared to the relevant background set of genes (Exp) in a subset of genes that excludes those in the bottom quartile of expression. Two-tailed *p*-values are defined as the probability of obtaining a mean as extreme as the observed only due to chance. Significant *p*-values after *FDR* correction for multiple tests are highlighted in bold (*FDR* < 0.05). The observed values of each parameter measured in the full set of sociality genes [Obs (all)] and the value expected from the full set of background genes [Exp (all)] are shown for comparison. *parameter values have been multiplied by 10^3.

Parameter	Exp (no low)	Obs (no low)	*p* (*FDR*)	Exp (all)	Obs (all)
π/site*	0.72	1.19	**<0.001**	0.73	1.21
π_a/site*	0.54	1.02	**<0.001**	0.55	1.04
π_s/site*	1.32	1.84	**<0.001**	1.33	1.84
Tajima's *D*	−0.64	−0.74	**<0.001**	−0.65	−0.71
K_a/K_s	0.17	0.24	**<0.001**	0.18	0.27
K_a*	0.87	1.41	**<0.001**	0.90	1.47
K_s*	6.95	9.05	**<0.001**	6.90	8.98
DoS	−0.018	−0.045	**0.045**	−0.022	−0.054
$D_n/(D_n + D_s)$	0.55	0.59	**0.002**	0.55	0.59
$P_n/(P_n + P_s)$	0.57	0.64	**<0.001**	0.58	0.65

Supplementary Table 11. Correlations between the index of social expression (ISE) and the set of evolutionary parameters that differ between sociality and the background set of genes. Two-tailed *p*-values are defined as the probability of obtaining a mean as extreme as the observed only due to chance. Significant *p*-values after *FDR* correction for multiple tests are highlighted in bold (*FDR* < 0.05).

Parameter	Correlation	*p* (*FDR*)
π/site	0.010	0.302
π_a/site	0.022	0.112
π_s/site	−0.018	0.136
K_a	−0.034	0.112
K_s	−0.023	0.153
$D_n/(D_n + D_s)$	0.014	0.302
$P_n/(P_n + P_s)$	0.019	0.136

Supplementary Table 12. Comparison of evolutionary parameters for conditional genes (observed) compared to those expected. Two-tailed *p*-values are defined as the probability of obtaining a mean as extreme as the observed only due to chance. Significant *p*-values after *FDR* correction for multiple tests are highlighted in bold (*FDR* < 0.05). For comparison the observed values of each parameter measured in the sociality genes and the value expected from the background set of genes used in the analysis of sociality genes are shown. *parameter values have been multiplied by 10^3.

Parameter	Exp (no low)	Obs (no low)	*p* (*FDR*)	Exp (all)	Obs (all)
π/site*	0.76	1.21	**<0.001**	0.73	1.21
π_a/site*	0.60	1.09	**<0.001**	0.55	1.04
π_s/site*	1.28	1.67	**<0.001**	1.33	1.84
Tajima's *D*	−0.62	−0.63	0.82	−0.65	−0.71
K_a/K_s	0.21	0.50	**<0.001**	0.18	0.27
K_a*	1.29	2.88	**<0.001**	0.90	1.47
K_s*	8.39	9.51	**<0.001**	6.90	8.98
DoS	−0.013	0.00030	0.44	−0.022	−0.054
$D_n/(D_n + D_s)$	0.56	0.65	**<0.001**	0.55	0.59
$P_n/(P_n + P_s)$	0.59	0.69	**<0.001**	0.58	0.65

Supplementayr Table 13. Complementary neutrality tests for social genes. Fu & Li's statistics compare external and internal branches of a genealogical tree. Under circumstances were variation is removed (purifying selection or recent selective sweeps), it is expected an excess of mutations in external branches (mutations segregating at low frequencies), resulting in negative values. Conversely, balancing selection maintains old alleles (inflating mutations in internal branches), resulting in positive values. Wall's *B* and *Q* statistics use linkage disequilibrium information to test whether a pair of segregating sites share the same genealogy – which would be inflated (larger values) under balancing selection. Expected values and the respective two-tailed *p*-values were obtained by a randomization process. For each group of social genes, we generated a set of 10,000 random groups of size *N* (where *N* is the number of genes in that particular group) sampled from a set that contains that group of social genes and its corresponding background set of genes. Two-tailed *p*-values are defined as the probability of obtaining a mean as extreme as the observed only due to chance after *FDR* correction for multiple tests. Values below 10^{-4} are presented as zeros.

Test	Group	Expected	Observed	*p* (*FDR*)
Fu & Li's *F**	Sociality	−0.733	−0.787	0.76
	Chimerism	−0.738	−0.713	0.90
	Antagonism	−0.733	−0.739	0.90
	Prespore	−0.739	−0.775	0.76
	Prestalk	−0.740	−0.704	0.76
	Presp-Prest	0.0006	−0.071	0.76
	Cheater	−0.733	−0.805	0.90
Fu & Li's *D**	Sociality	−0.638	−0.678	0.76
	Chimerism	−0.645	−0.605	0.90
	Antagonism	−0.637	−0.638	0.99
	Prespore	−0.638	−0.665	0.76
	Prestalk	−0.638	−0.611	0.76
	Presp-Prest	0.0006	−0.054	0.76
	Cheater	−0.638	−0.676	0.90
Wall's *B*	Sociality	0.0832	0.0833	0.99
	Chimerism	0.0822	0.0715	0.76
	Antagonism	0.0833	0.0790	0.76
	Prespore	0.0790	0.0777	0.90
	Prestalk	0.0790	0.0802	0.90
	Presp-Prest	0	−0.0025	0.90
	Cheater	0.0827	0.1022	0.76
Wall's *Q*	Sociality	0.113	0.113	0.99
	Chimerism	0.112	0.094	0.76
	Antagonism	0.113	0.107	0.76
	Prespore	0.107	0.105	0.90
	Prestalk	0.107	0.109	0.90
	Presp-Prest	0	−0.004	0.90
	Cheater	0.112	0.132	0.76

Supplementary Table 14. Enrichment analysis of social genes evolving under balancing selection as defined by different cutoffs of Tajima's *D*. We used a randomization procedure to test whether each of the groups of social genes contained an excess of genes evolving under balancing selection. For each group of social genes, we generated a set of 10,000 random groups of size *N* (where *N* is the number of genes in that particular group) sampled from a set that contains that group of social genes and its corresponding background set of genes. In each randomization we counted the number of genes evolving under balancing selection and used the distribution of the counts across randomizations to calculate the confidence intervals (2.5^{th} to 97.5^{th} percentiles).

Sites	Group	Observed	CI		*p* (*FDR*)
		**Tajima's *D* > 2**			
CDS	Sociality	13	5	16	> 0.05
	Chimerism	1	0	4	> 0.05
	Antagonism	12	7	20	> 0.05
	Prespore	5	2	12	> 0.05
	Prestalk	7	3	12	> 0.05
	Cheater	1	0	3	> 0.05
Nonsynonymous	Sociality	14	5	16	> 0.05
	Chimerism	1	0	4	> 0.05
	Antagonism	7	7	20	> 0.05
	Prespore	2	2	12	> 0.05
	Prestalk	5	3	12	> 0.05
	Cheater	1	0	3	> 0.05
Synonymous	Sociality	11	5	16	> 0.05
	Chimerism	0	0	4	> 0.05
	Antagonism	12	8	21	> 0.05
	Prespore	8	3	12	> 0.05
	Prestalk	4	2	12	> 0.05
	Cheater	1	0	3	> 0.05
		**Tajima's *D* > 1.5**			
CDS	Sociality	40	26	47	> 0.05
	Chimerism	2	1	9	> 0.05
	Antagonism	47	36	60	> 0.05
	Prespore	22	15	32	> 0.05
	Prestalk	25	16	34	> 0.05
	Cheater	1	0	6	> 0.05
Nonsynonymous	Sociality	40	28	50	> 0.05
	Chimerism	3	1	10	> 0.05
	Antagonism	42	38	62	> 0.05
	Prespore	14	15	33	> 0.05
	Prestalk	28	17	35	> 0.05
	Cheater	1	0	6	> 0.05
Synonymous	Sociality	51	36	60	> 0.05
	Chimerism	8	3	13	> 0.05
	Antagonism	68	52	80	> 0.05
	Prespore	32	23	44	> 0.05
	Prestalk	36	23	44	> 0.05
	Cheater	1	0	7	> 0.05
		**Tajima's *D* > 1**			

CDS	Sociality	72	61	93	> 0.05
	Chimerism	6	5	17	> 0.05
	Antagonism	94	83	117	> 0.05
	Prespore	45	36	61	> 0.05
	Prestalk	49	39	66	> 0.05
	Cheater	1	1	9	> 0.05
Nonsynonymous	Sociality	71	62	93	> 0.05
	Chimerism	10	5	17	> 0.05
	Antagonism	91	81	116	> 0.05
	Prespore	34	35	60	> 0.05
	Prestalk	57	38	64	> 0.05
	Cheater	3	1	10	> 0.05
Synonymous	Sociality	111	76	109	> 0.05
	Chimerism	13	8	22	> 0.05
	Antagonism	131	108	146	> 0.05
	Prespore	62	50	78	> 0.05
	Prestalk	69	49	77	> 0.05
	Cheater	3	2	11	> 0.05

Supplementary Table 15. Intraspecific variation in sociality genes excluding 13 genes evolving under balancing selection. Expected values and the respective two-tailed *p*-values were obtained from randomization distributions. We generated a set of 10,000 random groups of size *N* (where *N* is the number of genes in that particular group) sampled from a set that contains that sociality genes and its corresponding background set of genes. Significant *p*-values after *FDR* correction for multiple tests are highlighted in bold (*FDR* < 0.05).

Sites	Estimator	Expected ($x10^{-3}$)	Observed ($x10^{-3}$)	*p* (*FDR*)
CDS	π/site	0.715	1.177	**$< 10^{-4}$**
	SNP/site	4.419	6.759	**$< 10^{-4}$**
Nonsynonymou	π/site	0.553	1.022	**$< 10^{-4}$**
	SNP/site	3.490	5.896	**$< 10^{-4}$**
Synonymous	π/site	1.274	1.766	**$< 10^{-4}$**
	SNP/site	7.670	9.785	**$< 10^{-4}$**

Supplementary Table 16. Enrichment analysis of social genes showing strong signatures of selection. We used a randomization procedure to test whether each of the five groups of social genes contained an excess of genes from these two categories. For each group of social genes, we generated a set of 10,000 random groups of size *N* (where *N* is the number of genes in that particular group) sampled from a set that contains that group of social genes and its corresponding background set of genes. In each randomization we counted the number of genes evolving under these forms of selection and used the distribution of the counts across randomizations to calculate the confidence intervals (2.5th to 97.5th percentiles). Significant *p*-values after *FDR* correction for multiple tests are highlighted in bold (*FDR* < 0.05).

Type of selection	Group	Observed	CI		*p* (*FDR*)
Purifying/Balancing	Sociality	13	6	18	> 0.05
	Chimerism	0	0	3	> 0.05
	Antagonism	10	4	15	> 0.05
	Prespore	8	1	9	> 0.05
	Prestalk	8	1	9	> 0.05
	Cheater	2	0	2	> 0.05
Positive	Sociality	1	2	11	**0.031**
	Chimerism	2	0	3	> 0.05
	Antagonism	9	3	13	> 0.05
	Prespore	6	1	8	> 0.05
	Prestalk	6	1	8	> 0.05
	Cheater	1	0	2	> 0.05

Supplementary Table 17. Comparison of evolutionary parameters at genes showing biased expression in either prestalk and prespore cells (with expression biases of 0.8 or 0.9 shown separately) relative to the background of all of the genes expressed in the same cells (corresponding to the values plotted in Figure 4). Two-tailed *p*-values are defined as the probability of obtaining a mean as extreme as the observed value due to chance. Significant *p*-values after *FDR* correction for multiple tests are highlighted in bold (*FDR* < 0.05). All parameter values have been multiplied by 10^3.

Type of selection	Group	Expected	Observed	*p* (*FDR*)
Bias = 0.8	π/site	0.730	0.956	**<0.001**
	π_a/site	0.567	0.771	**<0.001**
	π_s/site	1.298	1.612	**<0.001**
	K_a/K_s	0.208	0.231	0.26
	K_a	0.976	1.250	**0.001**
	K_s	6.771	8.013	**<0.001**
Bias = 0.9	π/site	0.730	1.046	**0.022**
	π_a/site	0.567	0.882	**0.022**
	π_s/site	1.297	1.655	**0.045**
	K_a/K_s	0.208	0.234	0.59
	K_a	0.978	1.263	0.15
	K_s	6.759	9.586	**0.001**

Supplementary Table 18. Evolutionary statistics for prespore and prestalk genes. Expected values and the respective two-tailed *p*-values were obtained from randomization distributions (sampled from a combination of prespore and prestalk genes). For each group of genes, we generated a set of 10,000 random groups of size *N* (where *N* is the number of genes in that particular group) sampled from a set that contains that group of prespore or prestalk genes and its corresponding background set of genes. Two-tailed *p*-values are defined as the probability of obtaining a mean as extreme as the observed only due to chance. Significant *p*-values after familywise *FDR* correction for multiple tests are highlighted in bold (*FDR* < 0.05). Values listed as zero have a true value less than 10^{-6}. *parameter values have been multiplied by 10^3.

Parameter	Group	Expected	Observed	*p* (*FDR*)
π/site	Prespore*	0.81	0.84	0.43
	Prestalk*	0.81	0.78	0.43
	Presp-Prest	0	6.02×10^{-5}	0.43
π_a/site	Prespore*	0.63	0.66	0.52
	Prestalk*	0.63	0.61	0.52
	Presp-Prest	0	4.99×10^{-6}	0.52
π_s/site	Prespore*	1.45	1.50	0.43
	Prestalk*	1.45	1.41	0.43
	Presp-Prest	0	4.65×10^{-5}	0.43
Tajima's *D* CDS	Prespore	−0.66	−0.70	0.27
	Prestalk	−0.66	−0.63	0.27
	Presp-Prest	0.14×10^{-3}	−0.073	0.27
Tajima's *D* Nsyn	Prespore	−0.67	−0.72	0.14
	Prestalk	−0.67	−0.62	0.14
	Presp-Prest	0.23×10^{-3}	−0.11	0.14
Tajima's *D* Syn	Prespore	−0.44	−0.47	0.43
	Prestalk	−0.44	−0.42	0.43
	Presp-Prest	0.15×10^{-3}	−0.051	0.43
DoS	Prespore	−0.029	−0.0435	0.33
	Prestalk	−0.029	−0.0154	0.33
	Presp-Prest	-0.63×10^{-6}	−0.0281	0.33
$P_n/(P_n+P_s)$	Prespore	0.585	0.585	0.98
	Prestalk	0.585	0.585	0.98
	Presp-Prest	-0.22×10^{-6}	0.36×10^{-3}	0.98
$D_n/(D_n+D_s)$	Prespore	0.552	0.536	0.27
	Prestalk	0.552	0.567	0.27
	Presp-Prest	-0.96×10^{-6}	−0.032	0.27
K_a/K_s	Prespore	0.194	0.212	0.27
	Prestalk	0.194	0.177	0.27
	Presp-Prest	3.2×10^{-5}	0.036	0.27
K_a	Prespore*	1.01	1.03	0.74
	Prestalk*	1.01	0.99	0.74
	Presp-Prest	0	4.1×10^{-5}	0.74
K_s	Prespore*	7.27	6.81	0.14
	Prestalk*	7.27	7.71	0.14
	Presp-Prest	0	9.1×10^{-4}	0.14

Supplementary Table 19. Enrichment analysis of the number of prespore and prestalk genes carrying at least one mutation that introduces a stop codon or results in a partial deletion (presence/absence variation). We used a randomization procedure to test whether each of the two groups of genes contained an excess of genes carrying these types of deleterious mutations. For each group of genes, we generated a set of 10,000 random groups of size *N* (where *N* is the number of genes in that particular group) sampled from a set that contains that group of social genes and its corresponding background set of genes. In each randomization we counted the number of genes that contained each type of deleterious mutation and used the distribution of the counts across randomizations to calculate the confidence intervals (2.5^{th} to 97.5^{th} percentiles) and *p*-values. Significant *p*-values after *FDR* correction for multiple tests are highlighted in bold (*FDR* < 0.05).

Class of mutations	Group	Observed	CI		*p* (*FDR*)
Stop codon gain	Prespore	1	1	8	> 0.05
	Prestalk	5	2	10	> 0.05
Presence/Absence	Prespore	0	3	13	**$< 10^{-3}$**
	Prestalk	0	4	14	**$< 10^{-3}$**

Supplementary Table 20. Genome sequencing statistics for the strains included in analyses. Strain IDs match those from the Dicty Stock Center. Five of the strains (marked with an asterisk) were sequenced in two independent libraries and for these, all statistics are for the pooled set of sequences from the two libraries (note that one strain, NC60.1, was sequenced using two different read lengths). For each strain the sequencing statistics given are: the total number of reads, raw read length (before any trimming), number of non-contaminant reads (which indicates the number of reads remaining after identified contaminant were removed), mapped reads (i.e., the number mapped to the reference genome), mapped read percentage (percentage of non-contaminant reads that mapped to the reference genome), per base mean coverage across the genome (including floating contigs, mitochondrial, and ribosomal chromosomes), and per base mean coverage across chromosomes (which is the average coverage per base across the 6 canonical chromosomes, excluding floating contigs, mitochondrial and ribosomal chromosomes). The last column ('Included in polymorph. analysis?') includes a 1 to indicate strains that were used in calculations of patterns of polymorphism and a zero for those excluded from that analysis.

Strain ID	Total reads	Read length	Non-contam. reads	Mapped reads	Mapped reads %	Per base mean coverage (genome)	Per base mean coverage (chrom.)	Included in polymorph. analysis?
B10	34385014	75	31432596	30871990	98.21	58.40	52.88	1
C5A	23321484	75	21031964	20324093	96.48	38.43	31.50	1
CT10B	20763216	100	16548320	16252852	98.21	40.99	11.32	1
DCB10B1	21411036	100	14473888	13465688	93.03	34.23	21.94	1
DD10	8924128	100	8059734	7962941	98.80	22.44	12.01	1
DD11	18066502	100	15298270	14975233	97.89	42.70	9.18	1
DD185*	32156550	100	24271648	23284644	95.93	61.80	42.56	1
DD61	12319758	100	11371264	11226723	98.73	31.72	18.40	1
FW1H1B	20061764	100	16825222	11629871	69.12	29.60	18.68	1
GC1A	20783966	100	14904262	14097970	94.59	35.80	16.77	1
K10*	40108102	100	33116674	27045169	81.67	68.52	37.93	1
MFD	42742286	75	40131250	39400116	98.14	74.34	65.07	1
ML	9637686	100	8790188	8474660	96.41	23.76	14.91	1
NC105.1*	17166114	100	16141950	14010535	86.80	40.40	23.98	1
NC28.1	13217886	100	11430062	7279115	63.68	18.90	11.42	1
NC34.1	10884346	100	9735146	9243103	94.95	26.03	14.55	1
NC34.2	11332308	100	10532664	9654075	91.66	27.18	16.72	1
NC39.1	10590568	100	9856900	9791834	99.34	27.66	13.95	1
NC43.1	12126882	100	10684330	10491980	98.20	29.66	15.17	1
NC47.2	19254528	100	16699050	11797028	70.64	29.55	15.60	1
NC52.3	11223948	100	10438546	8304541	79.56	23.43	12.13	1
NC54.2	11641348	100	10706850	7102010	66.33	19.95	11.21	1
NC58.1	11262548	100	10457086	7533934	72.05	20.95	10.77	1
NC59.2	24165384	100	19375144	15117494	78.03	38.07	21.62	1
NC60.1*	64204148	100/75	58140654	50994639	87.71	100.29	85.29	1
NC60.2	11800386	100	11070524	8230005	74.34	22.56	12.20	1

NC61.1	27936808	100	22116714	16608045	75.09	40.98	30.80	1
NC63.2	13075110	100	11763250	9255376	78.68	25.93	14.04	1
NC66.2	17673410	100	11429722	8188917	71.65	20.89	11.59	1
NC67.2	15551052	100	14308188	14196947	99.22	40.14	21.47	1
NC69.1	11060934	100	9973384	9795018	98.21	27.56	12.38	1
NC70.1	23111816	100	16789088	16285599	97.00	41.58	27.19	1
NC71.1	9390808	100	8613060	8522557	98.95	24.04	12.94	1
NC73.1	13429248	100	12609366	7674711	60.87	19.98	12.63	1
NC75.2	10751628	100	6715160	5318106	79.20	13.40	9.26	1
NC76.1	11718310	100	10807784	9630468	89.11	26.95	15.30	1
NC77.1	27577534	100	18574722	17687435	95.22	44.77	24.06	1
NC78.2	11962440	100	11216942	10598239	94.48	29.87	16.28	1
NC80.1	11290350	100	10425402	9836757	94.35	27.75	16.67	1
NC85.2*	35375420	100	28070750	21157814	75.37	56.81	36.39	1
NC87.1	9525944	100	8656322	8567125	98.97	24.19	12.99	1
NC88.2	10576868	100	9837448	9712723	98.73	27.39	14.10	1
NC94.1	20262894	100	16165914	15710518	97.18	39.92	25.60	1
NC96.1	11221844	100	10495372	9721503	92.63	27.45	13.86	1
NC98.1	20486210	100	16809728	15732687	93.59	40.22	22.14	1
NC99.1	10186228	100	9348362	8316244	88.96	23.02	13.28	1
QS102*	37899498	100	27167974	25137071	92.52	66.45	34.25	1
QS111	17479192	100	15348940	11487780	74.84	31.11	12.36	1
QS112	21527728	100	12769174	8745058	68.49	24.98	4.63	1
QS31	10476544	100	9444146	7650527	81.01	21.45	12.89	1
S109*	43936088	100	27080588	19621561	72.46	51.27	18.15	1
S210	27993674	75	19145660	14858477	71.24	27.99	19.23	1
S212	29509580	100	20969382	19998803	95.37	48.35	11.65	1
S220	12037102	100	10766150	10480091	97.34	29.62	15.91	1
S224	24590136	100	20886932	20409167	97.71	51.27	28.07	1
S63	22773340	100	12571660	10373749	82.52	26.22	17.44	1
SB42A	26052828	75	22570436	21740206	96.31	41.03	34.03	1
V12	26079132	75	23167030	22621241	97.61	42.78	34.01	1
WS10	17377982	100	14390562	14068133	97.76	39.95	10.97	1
WS269	30659418	75	20002946	18520159	92.08	34.93	28.96	1
WS380B	20617846	75	16973572	15375271	90.14	29.01	22.84	1
WS51	46376390	75	40429304	38927788	96.27	73.28	59.35	1
WS576	44416436	75	38170062	36656555	96.01	69.16	54.44	1
WS582	15720948	75	14782818	14467251	97.80	27.18	22.11	1
WS583	33805698	75	23190242	21675680	93.39	40.85	28.90	1
WS655	44048228	75	38063298	34875910	88.36	65.79	54.92	1
WS656	26786176	75	21880706	20908903	95.49	39.41	26.01	1
CF3B	31266072	75	27950312	21726675	75.93	38.63	36.35	0
CRII6C	16589978	100	11964168	10445830	87.31	25.92	19.81	0
KGL29A	14915434	100	7941930	5778992	72.77	13.86	12.21	0
OT3A	10082296	100	9208118	8147544	88.48	21.75	13.36	0
QS105	21711416	100	16959584	11654253	68.72	31.34	13.03	0

S6B	24350904	100	20829444	17766146	85.29	43.87	34.75	0
TAB13B	22494010	100	14591716	10746610	73.65	27.16	14.87	0
V34	41029130	75	35847442	28754674	78.66	51.32	45.54	0
WS205	106845752	75	38732084	36773302	94.91	69.01	53.42	0
WS2054	57867168	75	50601032	49038594	96.87	91.92	78.27	0
WS2162	39042866	75	32611428	31054375	95.21	58.51	49.99	0
WS584	32986834	75	11219322	3048226	21.23	4.98	3.08	0

Supplementary References

1. Rosengarten, R. D. *et al.* Leaps and lulls in the developmental transcriptome of Dictyostelium discoideum. *BMC Genomics* **16**, 294 (2015).

2. Noh, S., Geist, K. S., Tian, X., Strassmann, J. E. & Queller, D. C. Genetic signatures of microbial altruism and cheating in social amoebas in the wild. *Proc. Natl. Acad. Sci.* 201720324 (2018). doi:10.1073/pnas.1720324115

3. Parikh, A. *et al.* Conserved developmental transcriptomes in evolutionarily divergent species. *Genome Biol.* **11**, R35 (2010).

4. Santorelli, L. A. *et al.* Facultative cheater mutants reveal the genetic complexity of cooperation in social amoebae. *Nature* **451**, 1107–1110 (2008).

5. Schilde, C. *et al.* A set of genes conserved in sequence and expression traces back the establishment of multicellularity in social amoebae. *BMC Genomics* **17**, 871 (2016).
