# Supplementary material for: Conditional expression explains molecular evolution of social genes in a microbe
Source: Nat Commun. 2019 Jul 23;10:3284. doi: 10.1038/s41467-019-11237-2 (PMC6650454; doi:10.1038/s41467-019-11237-2)
Supplement: Supplementary file 2 — Reporting Summary [file 41467_2019_11237_MOESM2_ESM.pdf]

nature research

Corresponding author(s): Jason Wolf & Chris Thompson

Last updated by author(s): May 23, 2019

Reporting Summary

Nature Research wishes to improve the reproducibility of the work that we publish. This form provides structure for consistency and transparency in reporting. For further information on Nature Research policies, see Authors & Referees and the Editorial Policy Checklist.

Statistics

For all statistical analyses, confirm that the following items are present in the figure legend, table legend, main text, or Methods section.

n/a	Confirmed	
☐	☒	The exact sample size (*n*) for each experimental group/condition, given as a discrete number and unit of measurement
☐	☒	A statement on whether measurements were taken from distinct samples or whether the same sample was measured repeatedly
☐	☒	The statistical test(s) used AND whether they are one- or two-sided<br>*Only common tests should be described solely by name; describe more complex techniques in the Methods section.*
☐	☒	A description of all covariates tested
☐	☒	A description of any assumptions or corrections, such as tests of normality and adjustment for multiple comparisons
☐	☒	A full description of the statistical parameters including central tendency (e.g. means) or other basic estimates (e.g. regression coefficient) AND variation (e.g. standard deviation) or associated estimates of uncertainty (e.g. confidence intervals)
☐	☒	For null hypothesis testing, the test statistic (e.g. *F*, *t*, *r*) with confidence intervals, effect sizes, degrees of freedom and *P* value noted<br>*Give P values as exact values whenever suitable.*
☒	☐	For Bayesian analysis, information on the choice of priors and Markov chain Monte Carlo settings
☒	☐	For hierarchical and complex designs, identification of the appropriate level for tests and full reporting of outcomes
☒	☐	Estimates of effect sizes (e.g. Cohen's *d*, Pearson's *r*), indicating how they were calculated

Our web collection on statistics for biologists contains articles on many of the points above.

Software and code

Policy information about availability of computer code

Data collection	No code was used for data collection
Data analysis	Sequence reads were cleaned for adapters and quality trimmed using Trimmomatic. Contamination of DNA from other species was removed by mapping to the list of reference genomes described in the manuscript and assigning them according to best mapping score using BBSplit, part of the BBMap package. SNP calling was performed using the Genome Analysis Toolkit GATK. PCR duplicates were marked using Picard tools. Evolutionary parameters were estimated for genes using the R package PopGenome. The pseudo genome of OT3A was built by inserting SNPs for this strain into the reference genome using VCFtools software package. CDSs for all genes were extracted using gffread61. Rates if substitutionswere estimated using R package seqinR. Reads were trimmed with skewer package. Reads were pseudo-aligned to transcripts of the D. discoideum reference genome using Kallisto64. Normalisation was performed using the TMM method implemented in edgeR. Estimates of expression were summarized to gene level by using sleuth. Data manipulation and all analyses of categorical variables were performed in R version 3.3.0 and RStudio version 0.99.902, using built-in functions. For all continuous variables, expected values were estimated using a linear model fitted using the mixed procedure in SAS 9.4.

For manuscripts utilizing custom algorithms or software that are central to the research but not yet described in published literature, software must be made available to editors/reviewers. We strongly encourage code deposition in a community repository (e.g. GitHub). See the Nature Research guidelines for submitting code & software for further information.

Data

Policy information about availability of data

All manuscripts must include a data availability statement. This statement should provide the following information, where applicable:

- Accession codes, unique identifiers, or web links for publicly available datasets
- A list of figures that have associated raw data
- A description of any restrictions on data availability

All data generated or used in the current study are publicly available. The list of genetic variants used in all analyses are available from the EMBL-EBI European

Variation Archive (EVA) (project: PRJEB28260 and analysis: ERZ681043). The transcriptome data used in the analysis of sociality genes were downloaded from NCBI Gene expression Omnibus (GEO: GSE61914). The list of prespore and prestalk genes used in the analysis of antagonism genes was obtained from ref. 7, which was combined with a list of all genes included in the original RNA-seq experiment from ref. 33. The list of cheater genes is available from ref. 38. The RNA-seq (transcriptome) data sets from the comparison of clonal and chimeric slugs (used in the analysis of conflict genes) are available from the NCBI Gene Expression Omnibus (GSE118081). The RNA-seq (transcriptome) data from the comparison of prestalk and presore regions (used to identify genes with biased expression in these regions for the linear model testing the effect of proportion of sociality genes) are available from the NCBI Sequence Read Archive (SRA) (accession: PRJNA543665).

Field-specific reporting

Please select the one below that is the best fit for your research. If you are not sure, read the appropriate sections before making your selection.

☐ Life sciences ☐ Behavioural & social sciences ☒ Ecological, evolutionary & environmental sciences

For a reference copy of the document with all sections, see nature.com/documents/nr-reporting-summary-flat.pdf

Ecological, evolutionary & environmental sciences study design

All studies must disclose on these points even when the disclosure is negative.

Study description	This study uses genome sequence data to compare signatures of molecular evolution across different classes of genes.
Research sample	This study focuses on the social amoeba Dictyostelium discoideum because of the nature of social interactions during development in this system. Genome sequence data were generated for all strains that were available from the Dicty Stock Center to provide the largest possible dataset for analyses.
Sampling strategy	Genome sequence data were generated for all strains that were available from the Dicty Stock Center to provide the largest possible dataset for analyses.
Data collection	Genome sequence data were generated by multiple facilities and were recorded digitally by the sequencing machines used in each case.
Timing and spatial scale	NA
Data exclusions	No data were excluded. Data were trimmed for quality purposes as described in the Methods.
Reproducibility	NA
Randomization	NA
Blinding	NA

Did the study involve field work? ☐ Yes ☒ No

Reporting for specific materials, systems and methods

We require information from authors about some types of materials, experimental systems and methods used in many studies. Here, indicate whether each material, system or method listed is relevant to your study. If you are not sure if a list item applies to your research, read the appropriate section before selecting a response.

Materials & experimental systems

n/a	Involved in the study
☒	☐ Antibodies
☒	☐ Eukaryotic cell lines
☒	☐ Palaeontology
☒	☐ Animals and other organisms
☒	☐ Human research participants
☒	☐ Clinical data

Methods

n/a	Involved in the study
☒	☐ ChIP-seq
☒	☐ Flow cytometry
☒	☐ MRI-based neuroimaging
